# Supplementary material for: Proteomics and bioinformatics analysis reveal potential roles of cadmium-binding proteins in cadmium tolerance and accumulation of Enterobacter cloacae
Source: PeerJ. 2019 Sep 2;7:e6904. doi: 10.7717/peerj.6904 (PMC6727835; doi:10.7717/peerj.6904)
Supplement: Supplemental Information 4 [file peerj-07-6904-s004.docx]

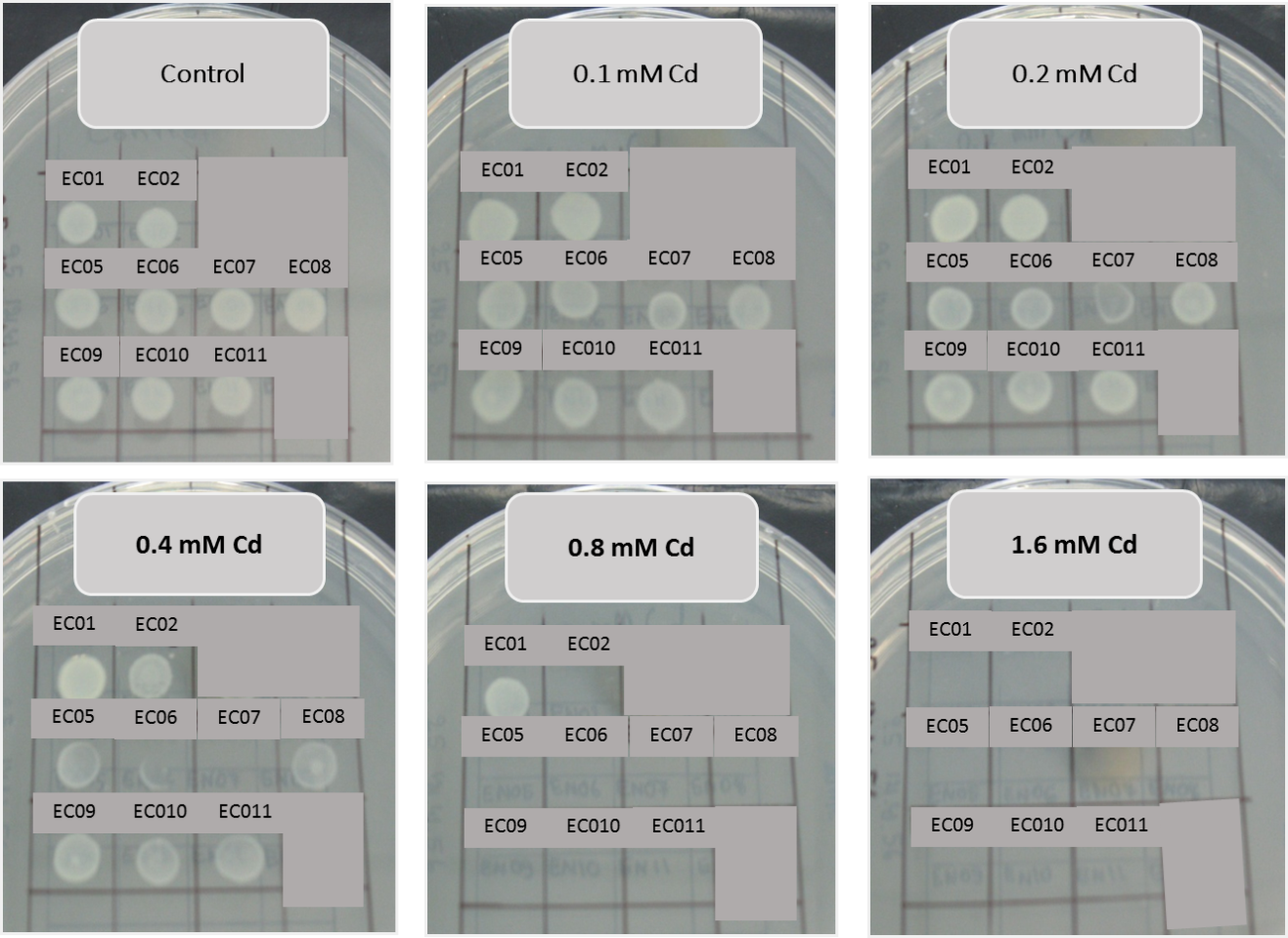


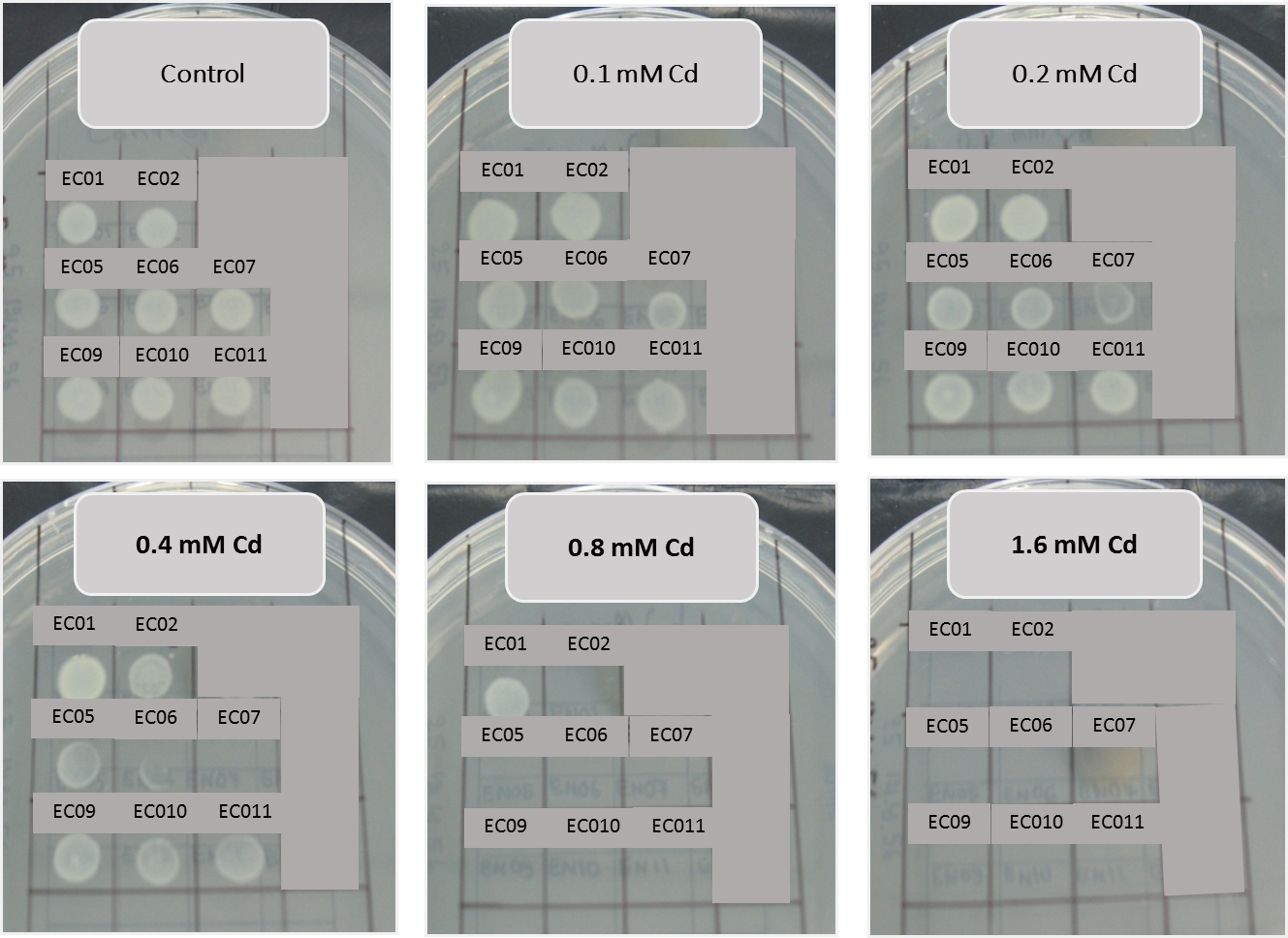


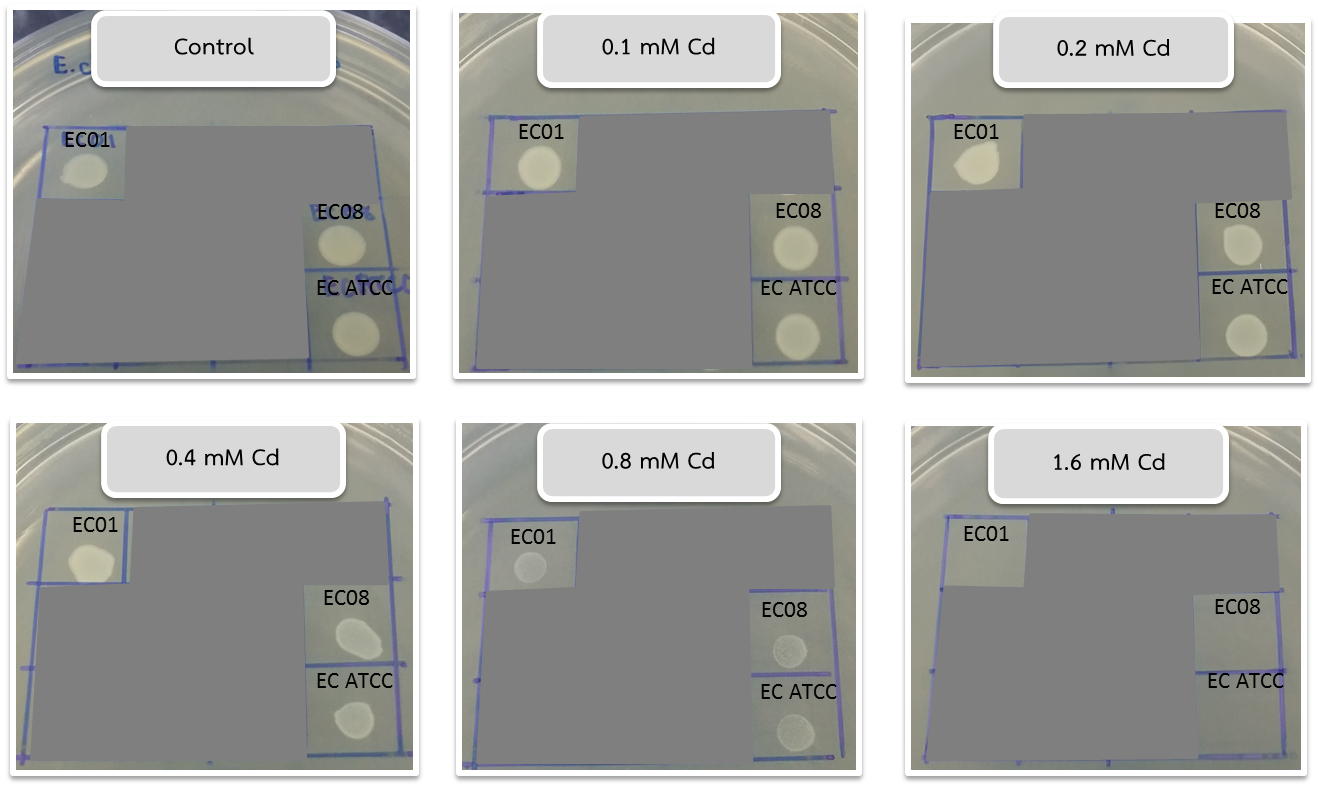


**Supplementary Figures 1**, **2** **and 3** The MICs of 9 isolates of *E. cloacae* and standard strain ATCC 13047 against cadmium were performed by the Agar Dilution method according to CLSI, 2012 recommendation


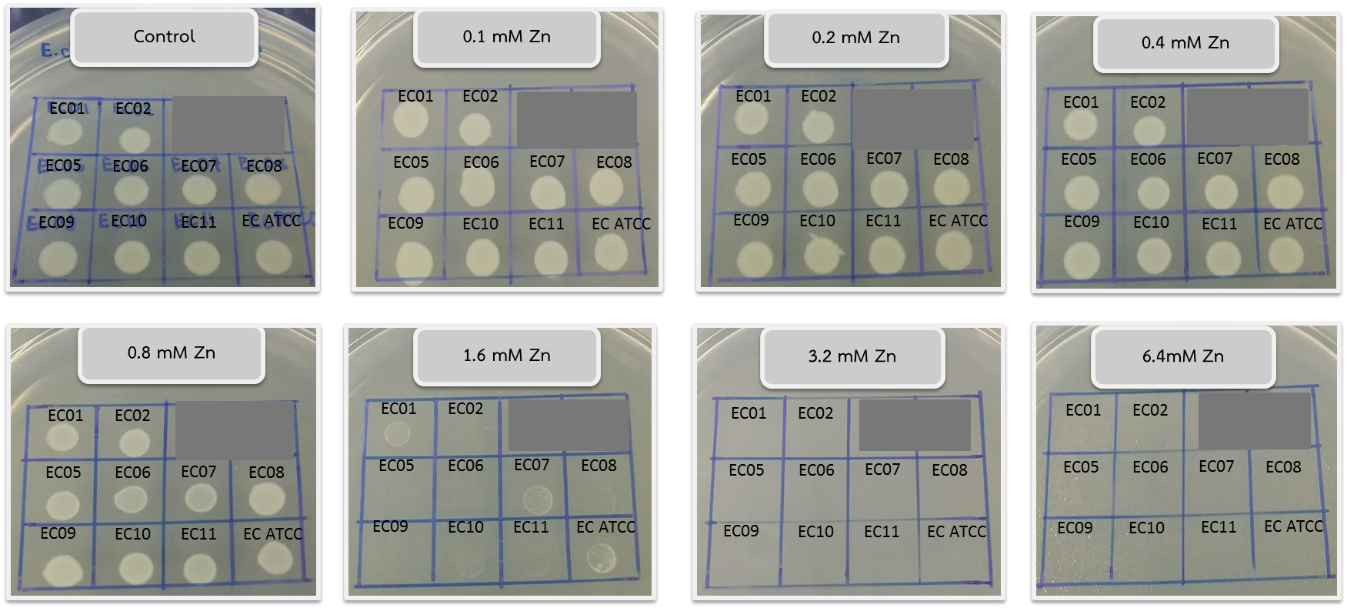


**Supplementary Figure 4** The MICs of 9 isolates of *E. cloacae* and standard strain ATCC 13047 against zinc were performed by the Agar Dilution method according to CLSI, 2012 recommendations


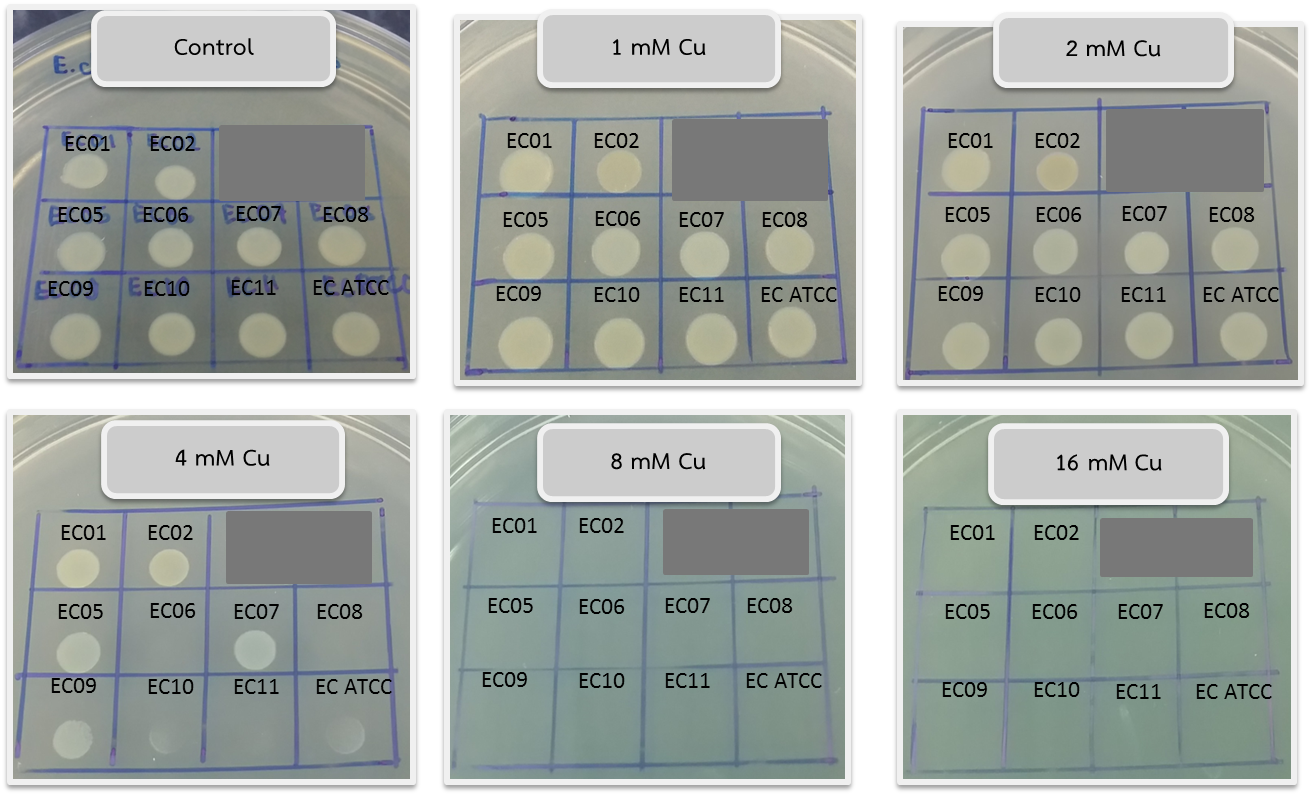


**Supplementary Figure 5** The MICs of 9 isolates of *E. cloacae* and standard strain ATCC 13047 against copper were performed by the Agar Dilution method according to CLSI, 2012 recommendations
