# Supplementary material for: Proteomics and bioinformatics analysis reveal potential roles of cadmium-binding proteins in cadmium tolerance and accumulation of Enterobacter cloacae
Source: PeerJ. 2019 Sep 2;7:e6904. doi: 10.7717/peerj.6904 (PMC6727835; doi:10.7717/peerj.6904)

Supplementary Table 1 2D-DIGE experimental design.

Gel	Cy2	Cy3	Cy5
**1**	Pooled Standard	07 Cd N1	ATCC Ctrl N1
**2**	Pooled Standard	01 Ctrl N2	01 Cd N2
**3**	Pooled Standard	01 Ctrl N1	07 Ctrl N3
**4**	Pooled Standard	07 Ctrl N2	07 Cd N2
**5**	Pooled Standard	ATCC Ctrl N3	07 Ctrl N1
**6**	Pooled Standard	07 Cd N3	01 Ctrl N3
**7**	Pooled Standard	ATCC Ctrl N2	ATCC Cd N2
**8**	Pooled Standard	ATCC Cd N1	01 Cd N1
**9**	Pooled Standard	01 Cd N3	ATCC Cd N3

Gel 1 Cy2 vs 07 Cd N1(Cy3) vs ATCC Ctrl N1(Cy5)

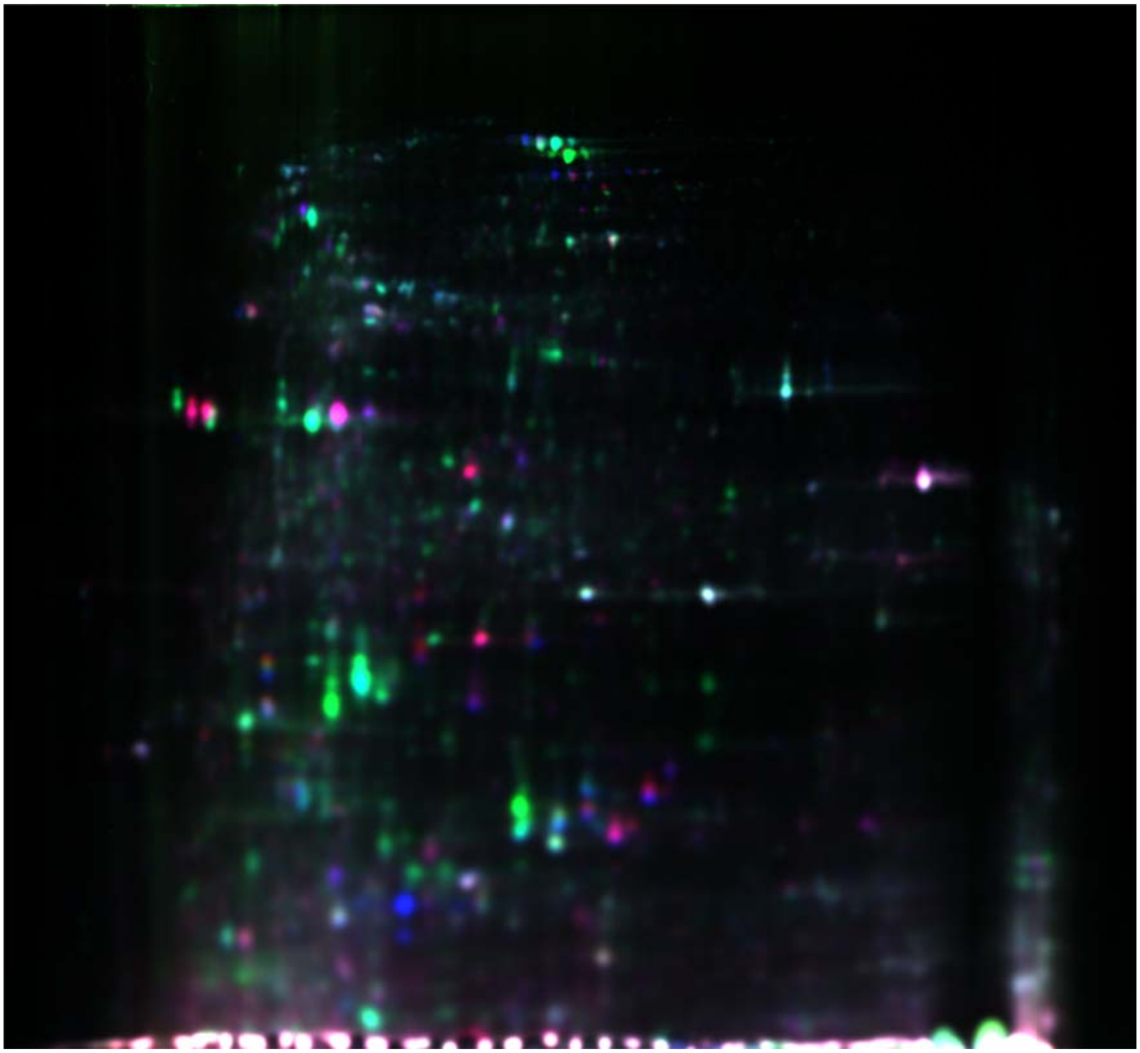

Gel 1 Cy2 vs 07 Cd N1(Cy3)

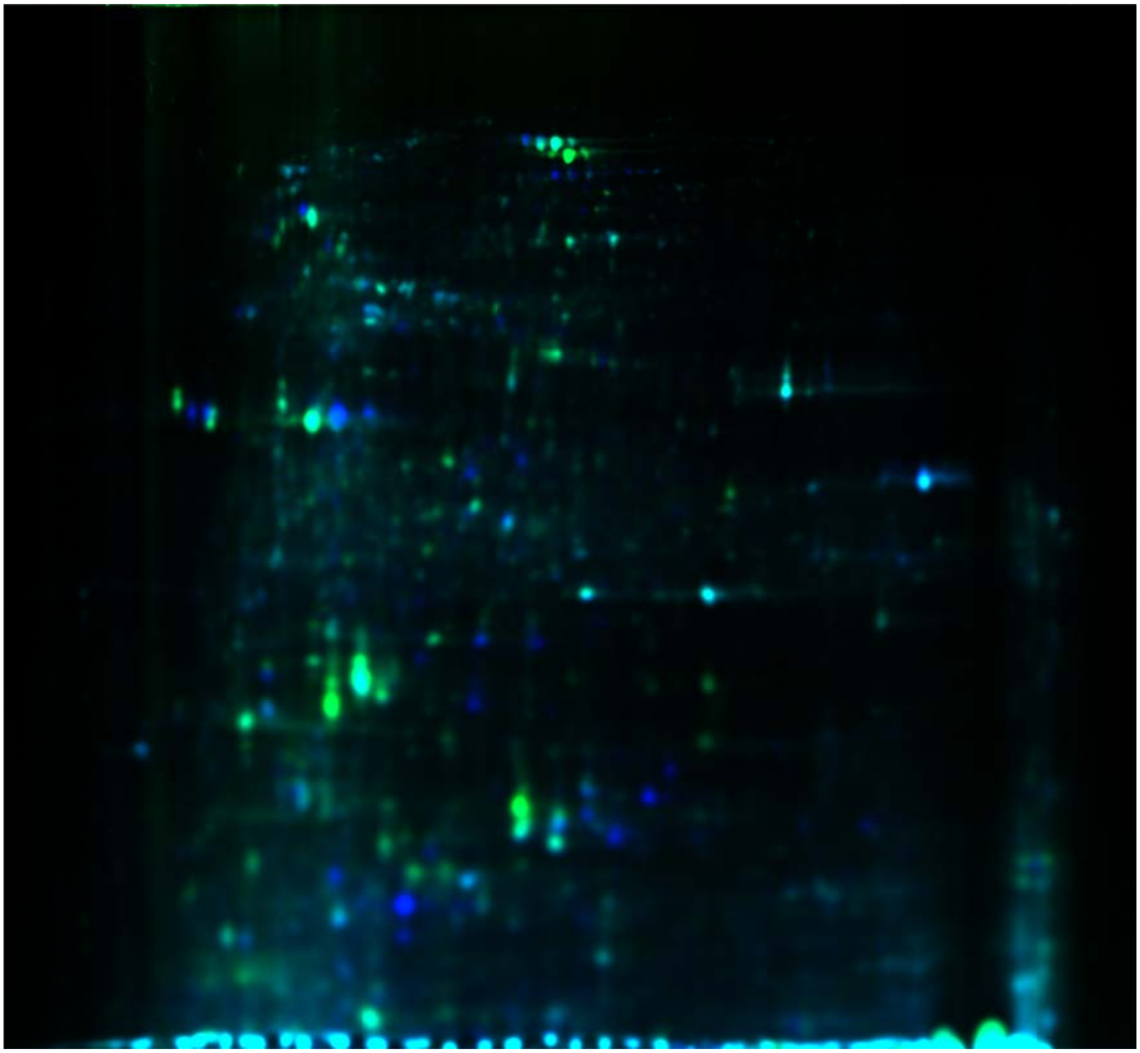

Gel 1 Cy2 vs ATCC Ctrl N1(Cy5)

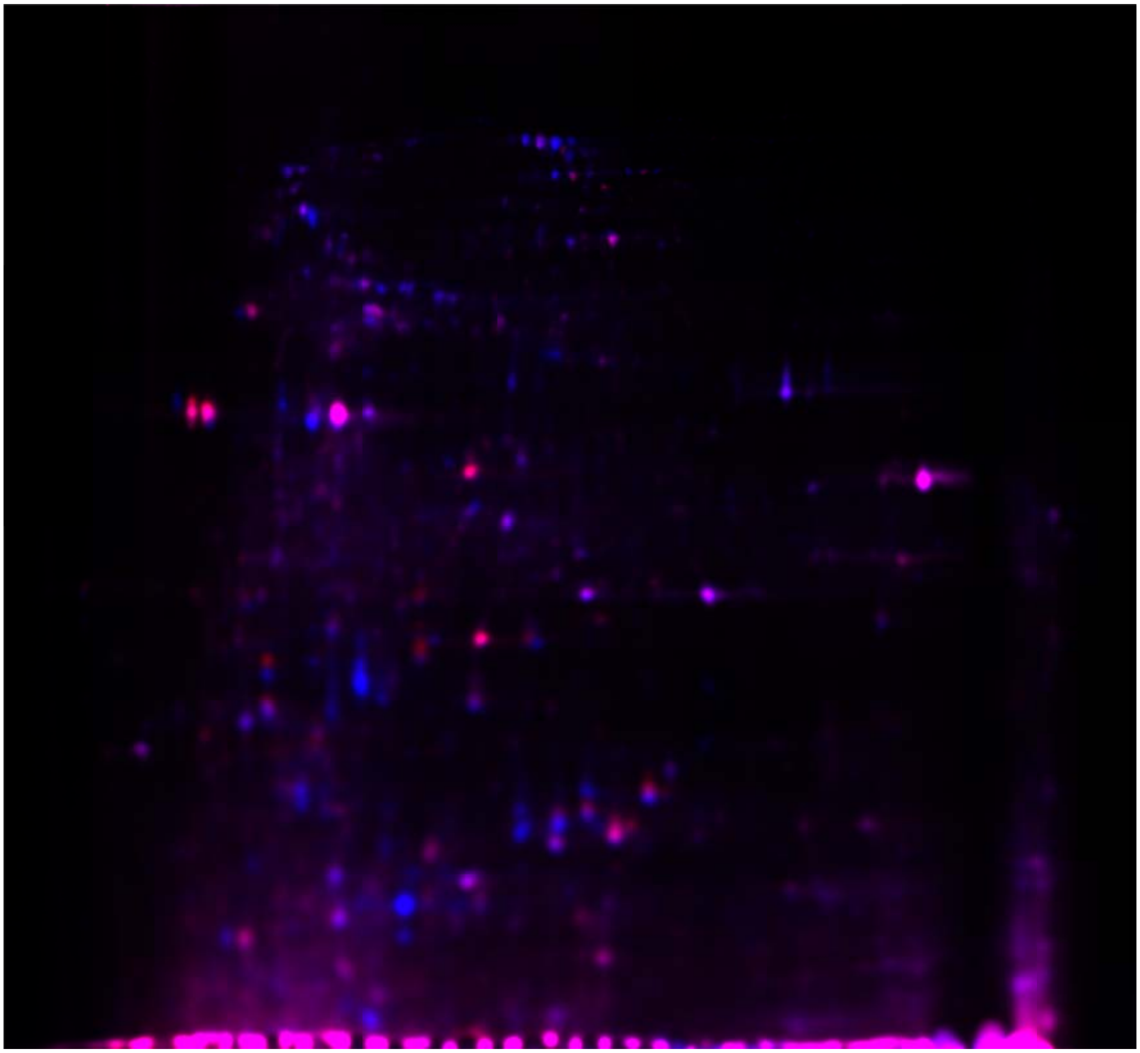

Gel 1 07 Cd N1(Cy3) vs ATCC Ctrl N1(Cy5)

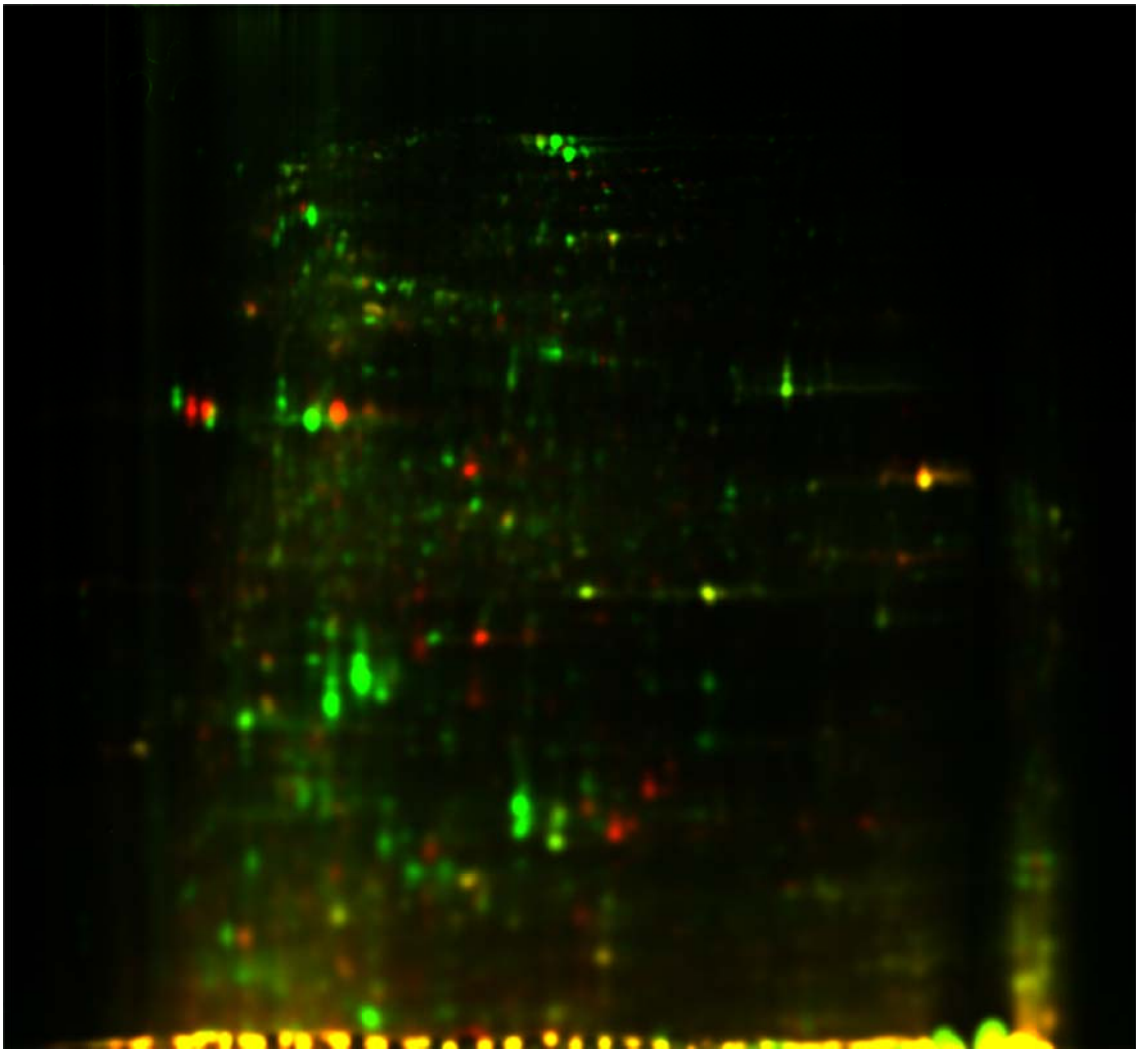

Gel 2 Cy2 vs 01 Ctrl N2(Cy3) vs 01 Cd N2(Cy5)

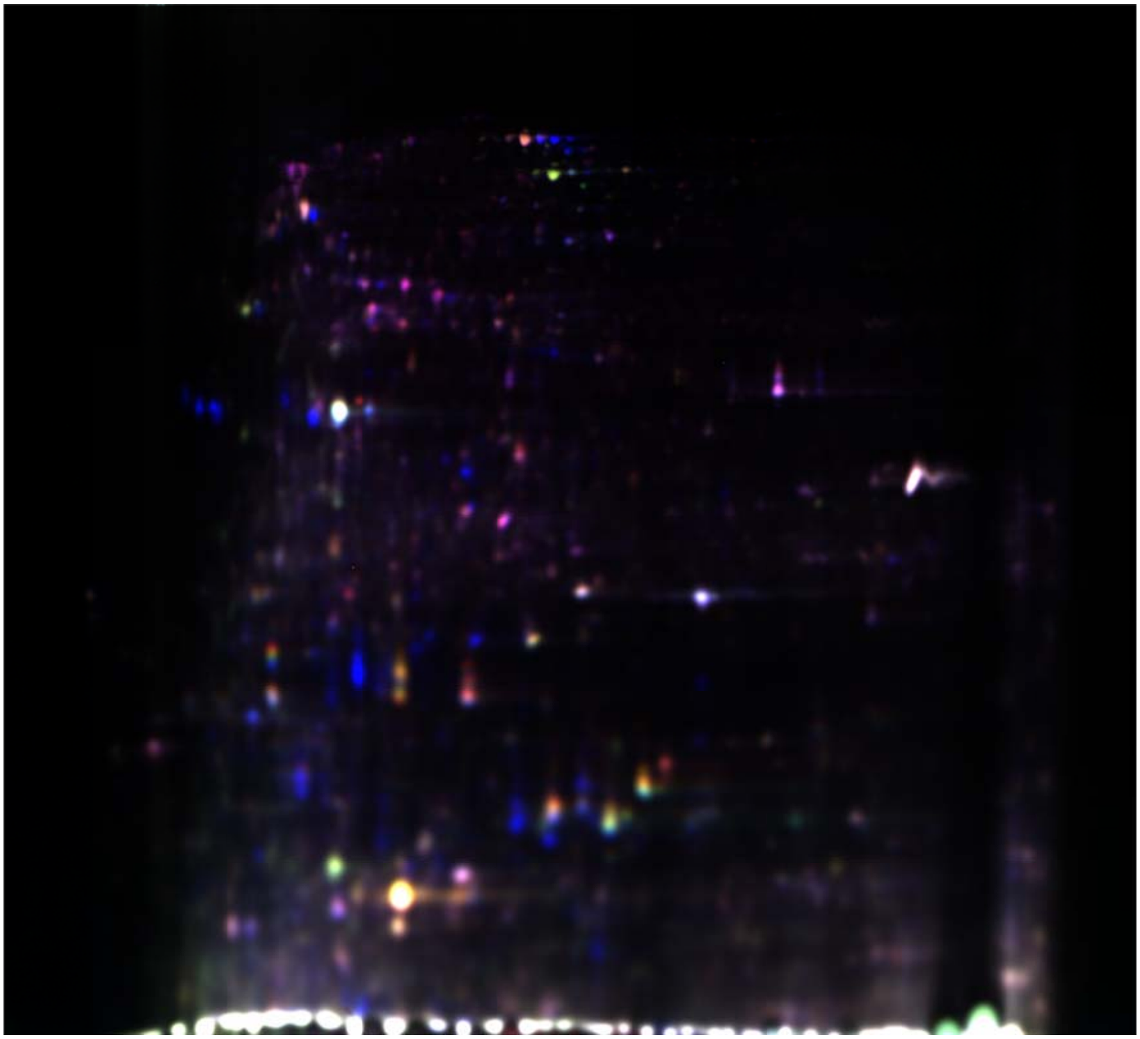

Gel 2 Cy2 vs 01 Ctrl N2(Cy3)

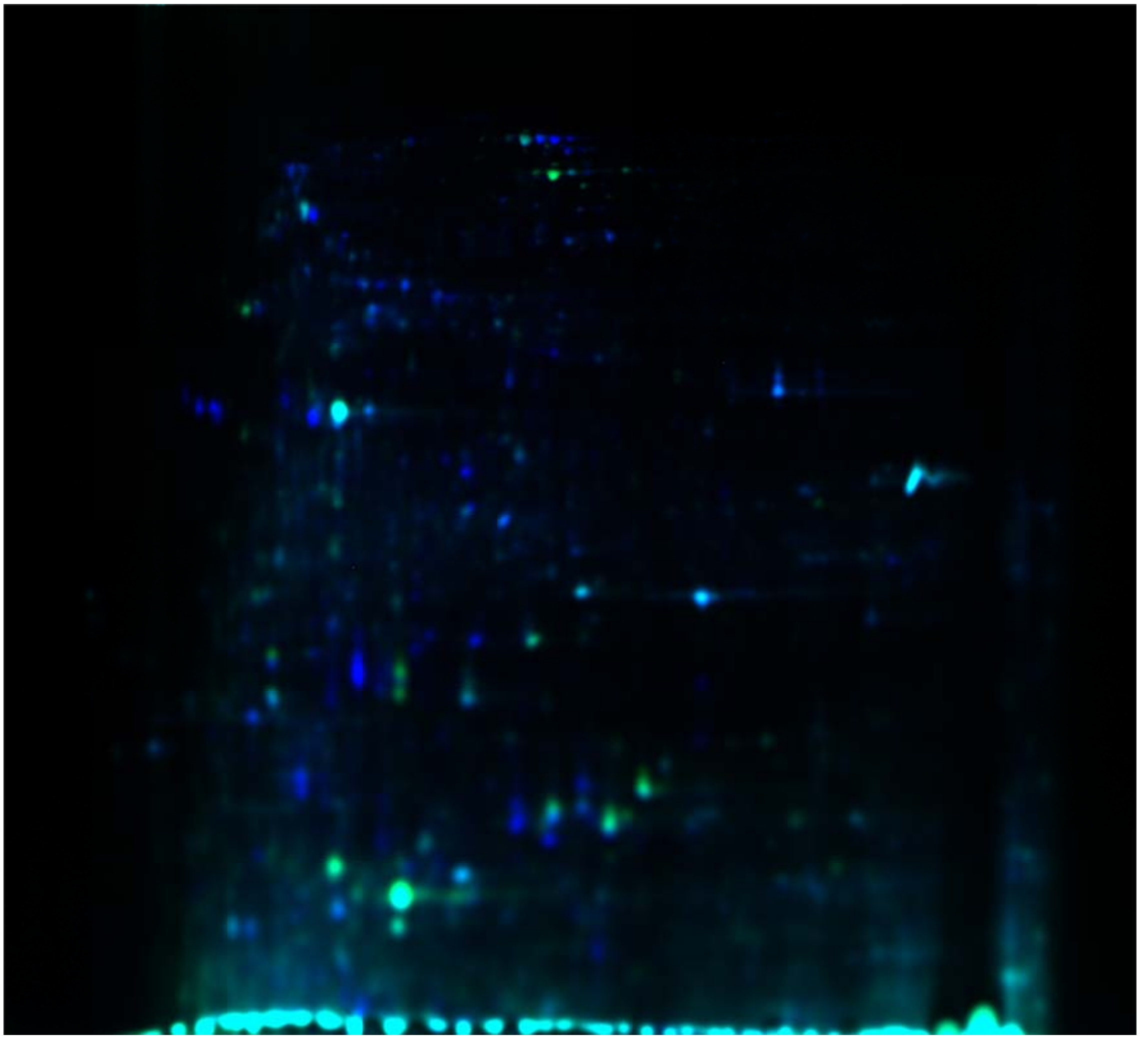

Gel 2 Cy2 vs 01 Cd N2(Cy5)

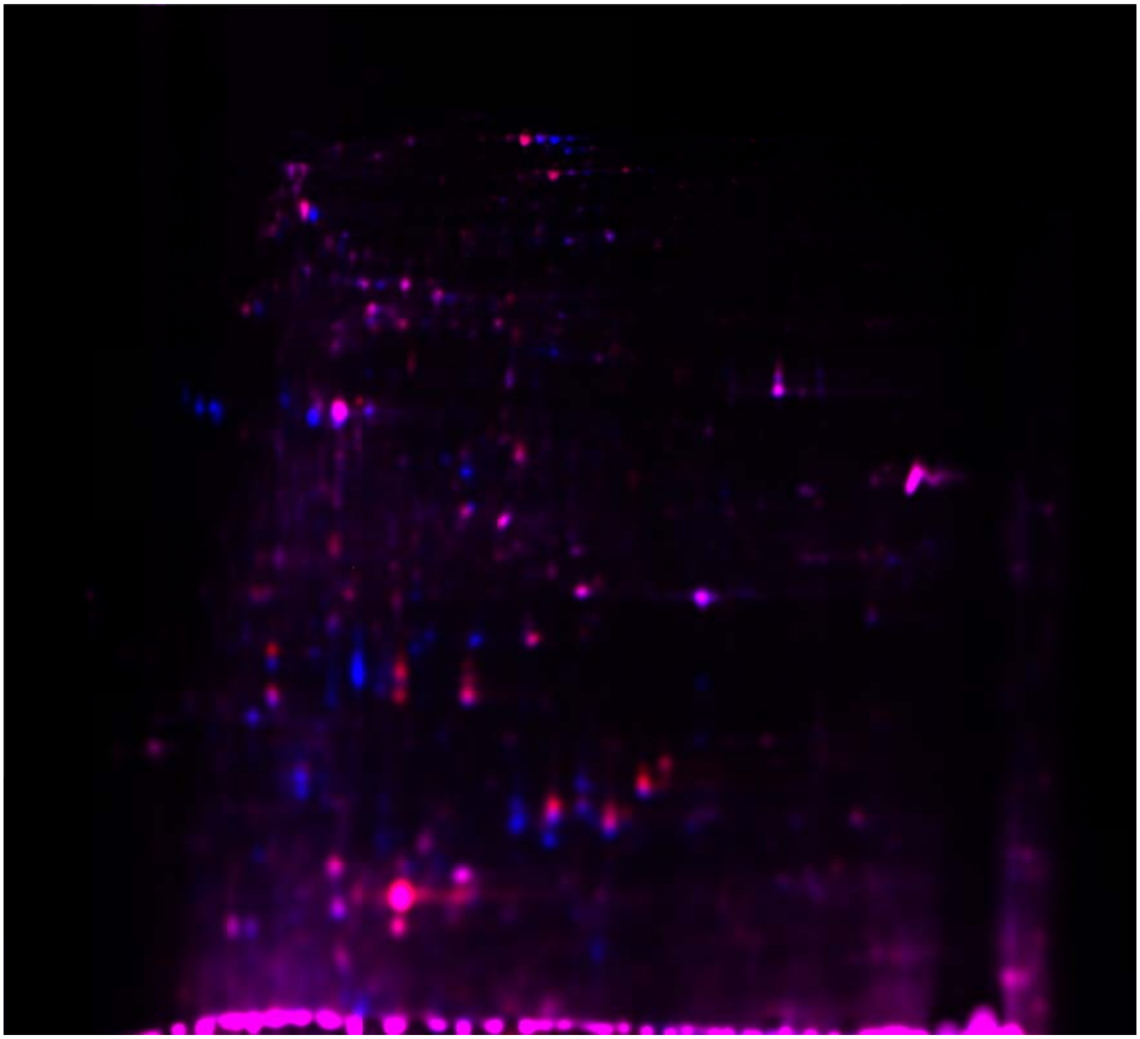

Gel 2 01 Ctrl N2(Cy3) vs 01 Cd N2(Cy5)

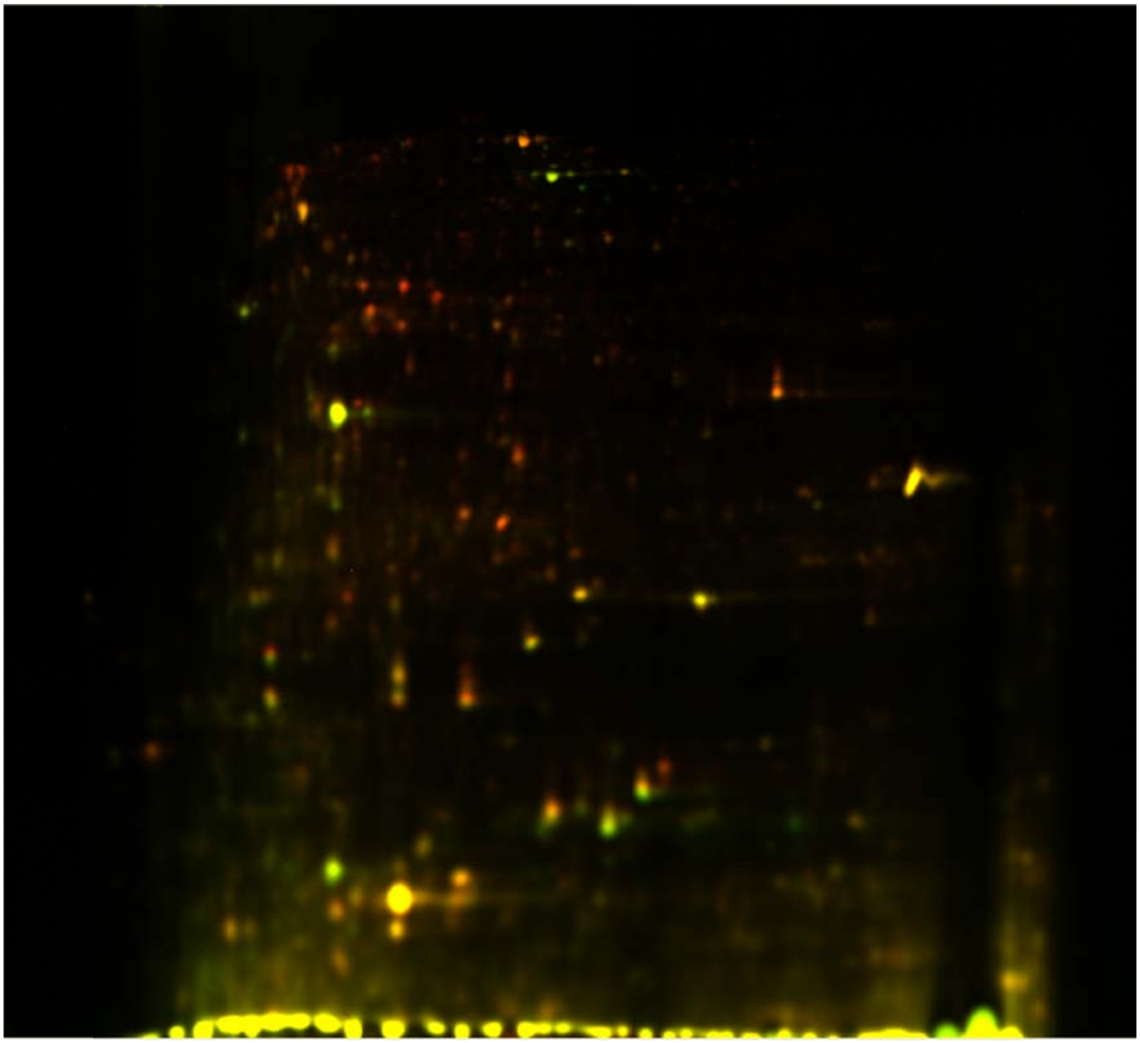

Gel 3 Cy2 vs 01 Ctrl N1(Cy3) vs 07 Ctrl N3(Cy5)

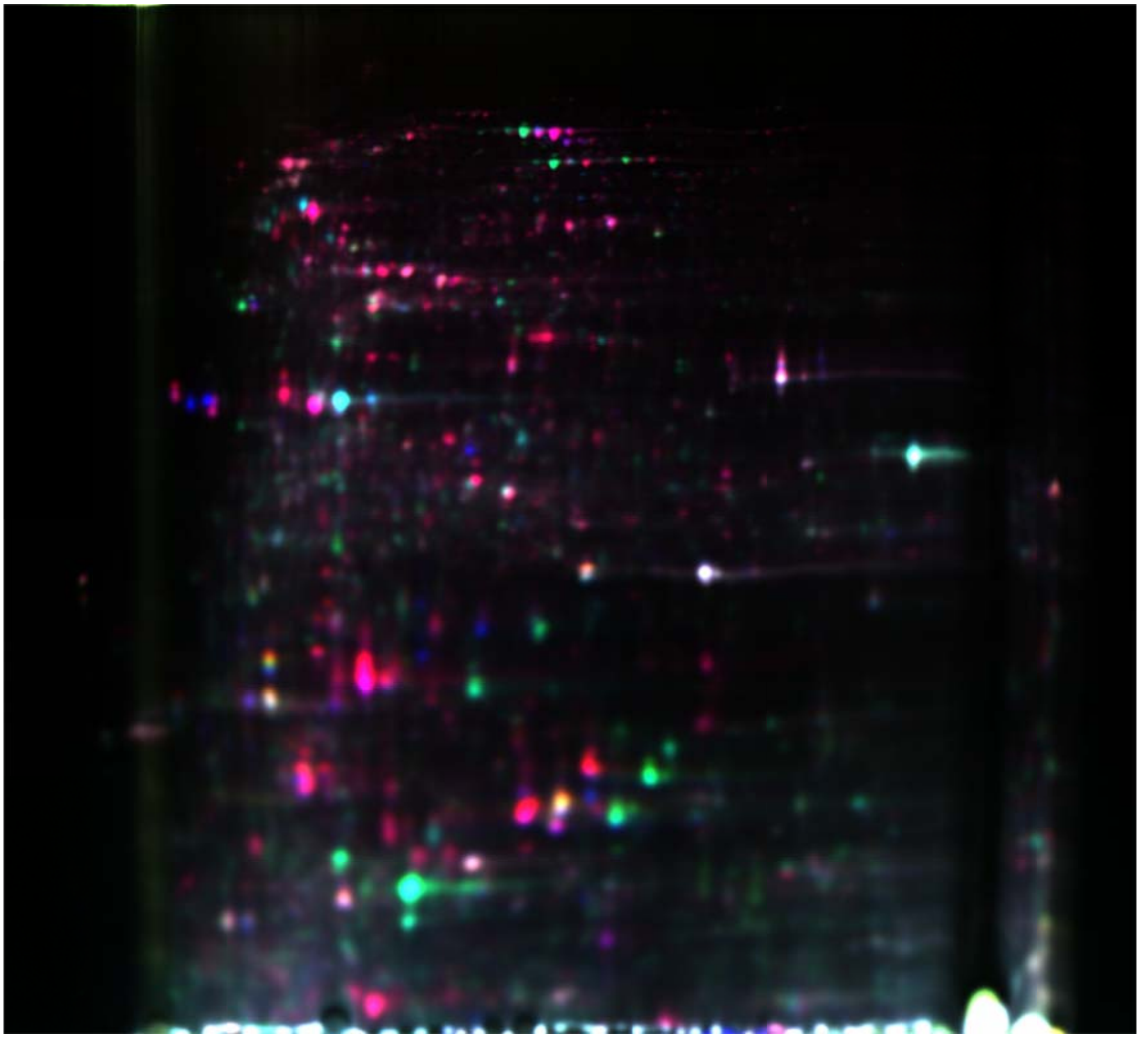

Gel 3 Cy2 vs 01 Ctrl N1(Cy3)

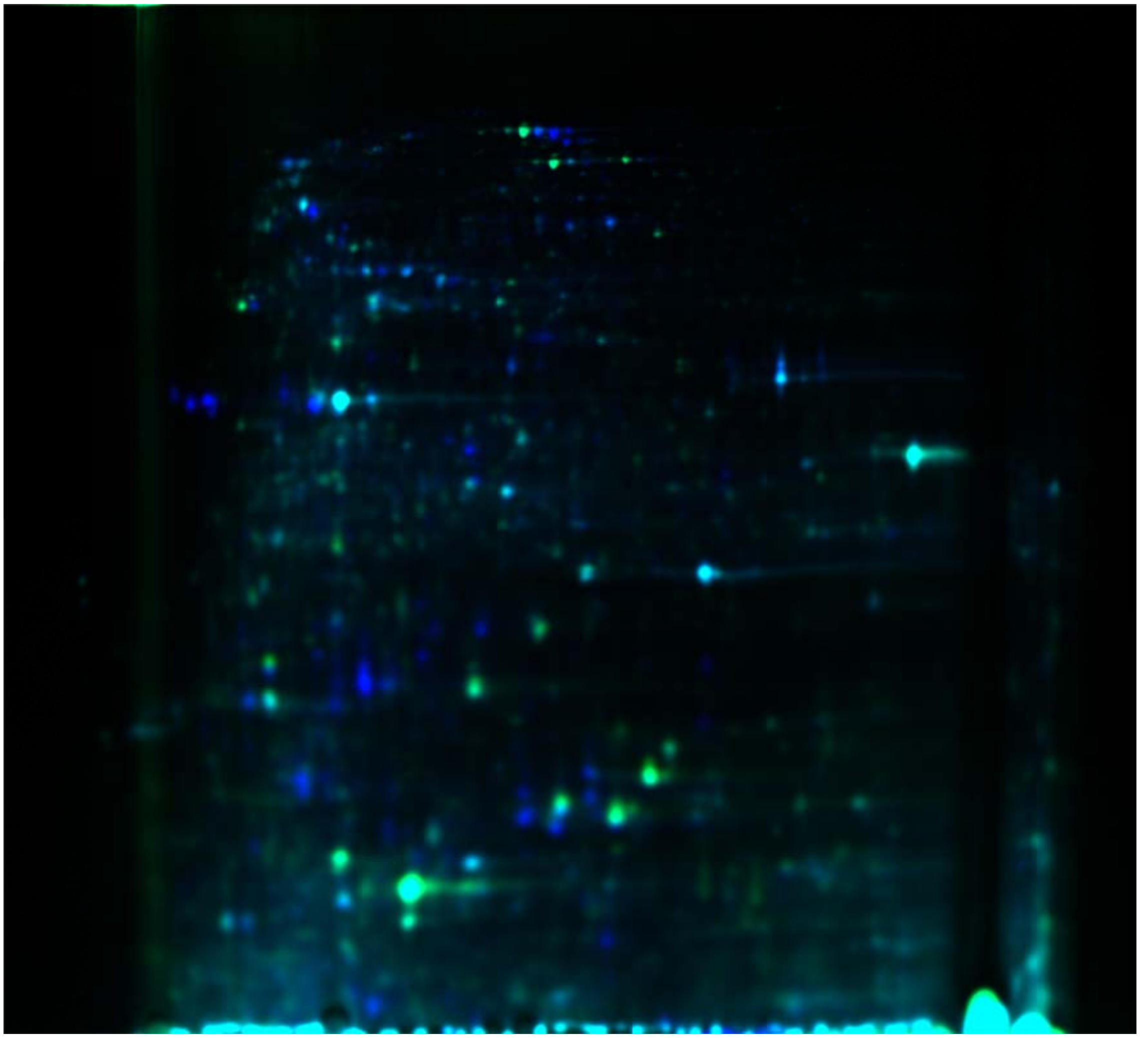

Gel 3 Cy2 vs 07 Ctrl N3(Cy5)

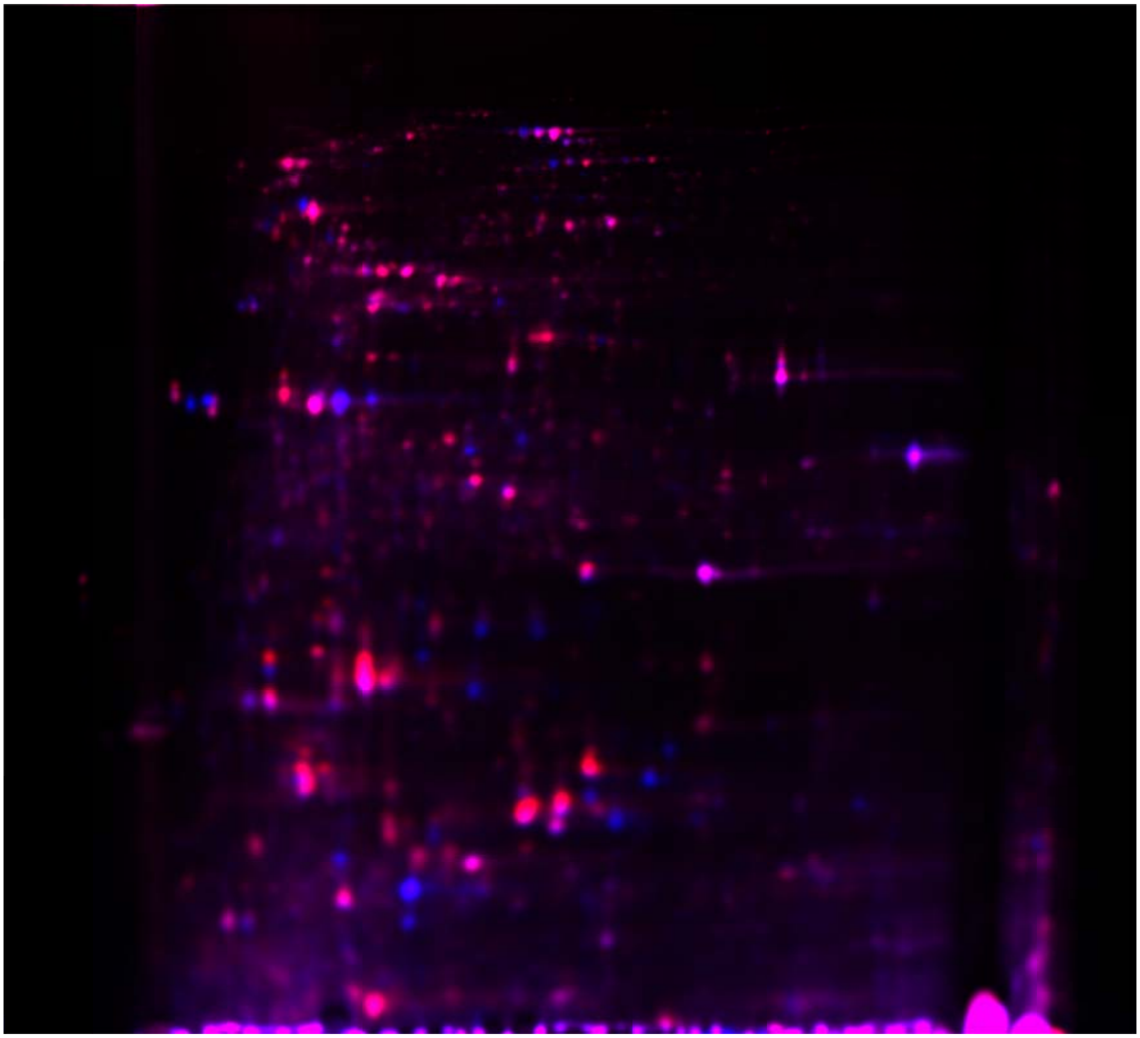

Gel 3 01 Ctrl N1(Cy3) vs 07 Ctrl N3(Cy5)

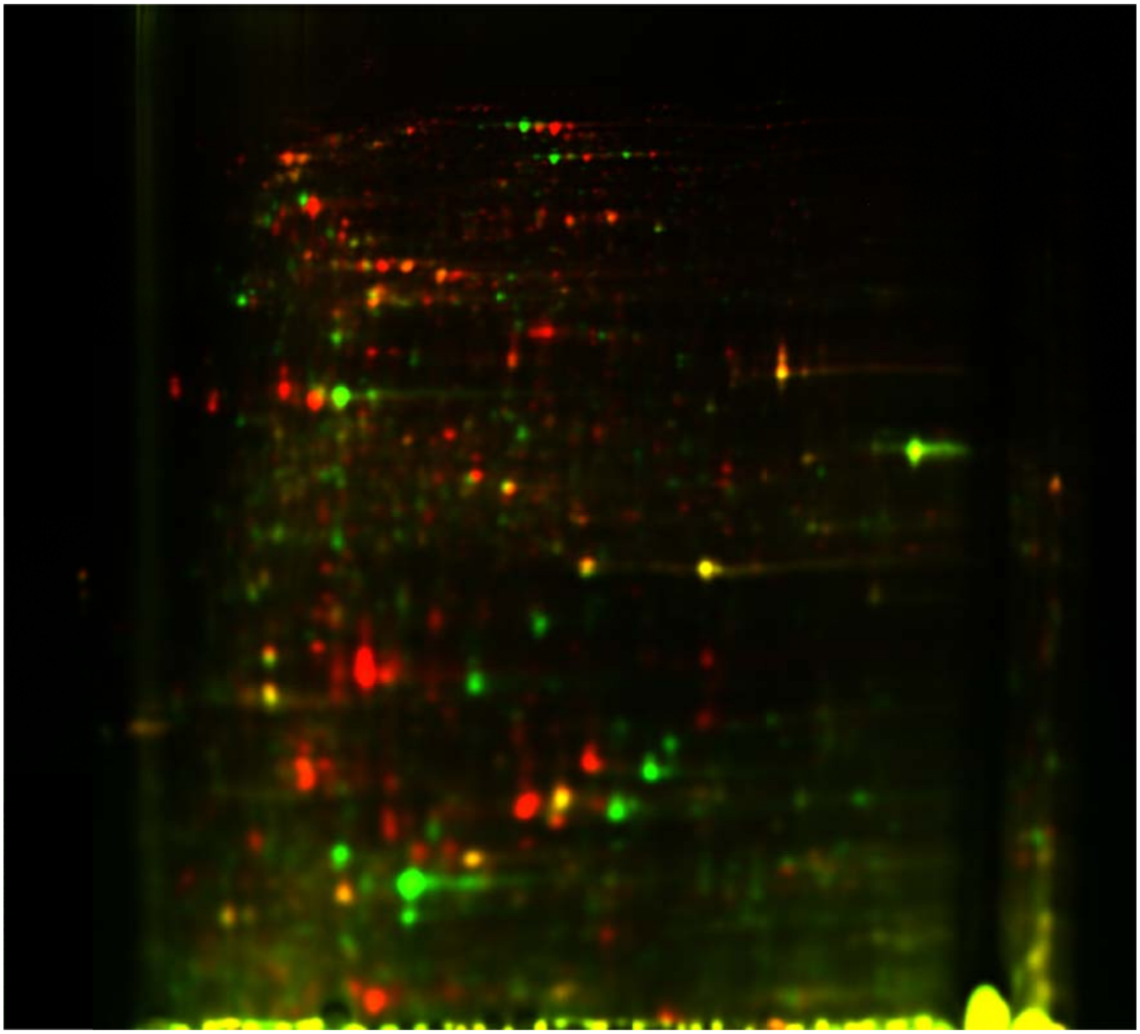

Gel 4 Cy2 vs 07 Ctrl N2(Cy3) vs 07 Cd N2(Cy5)

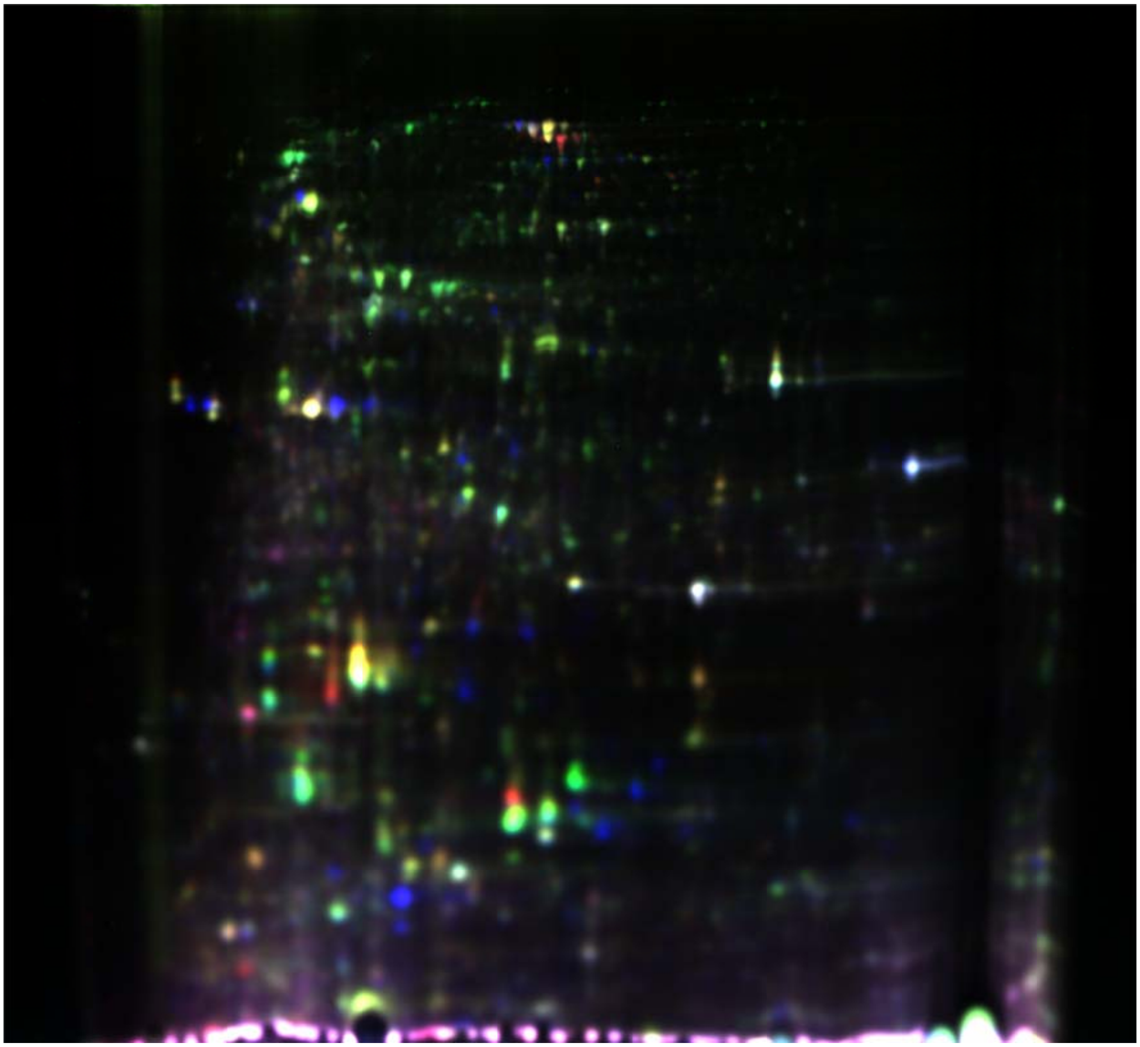

Gel 4 Cy2 vs 07 Ctrl N2(Cy3)

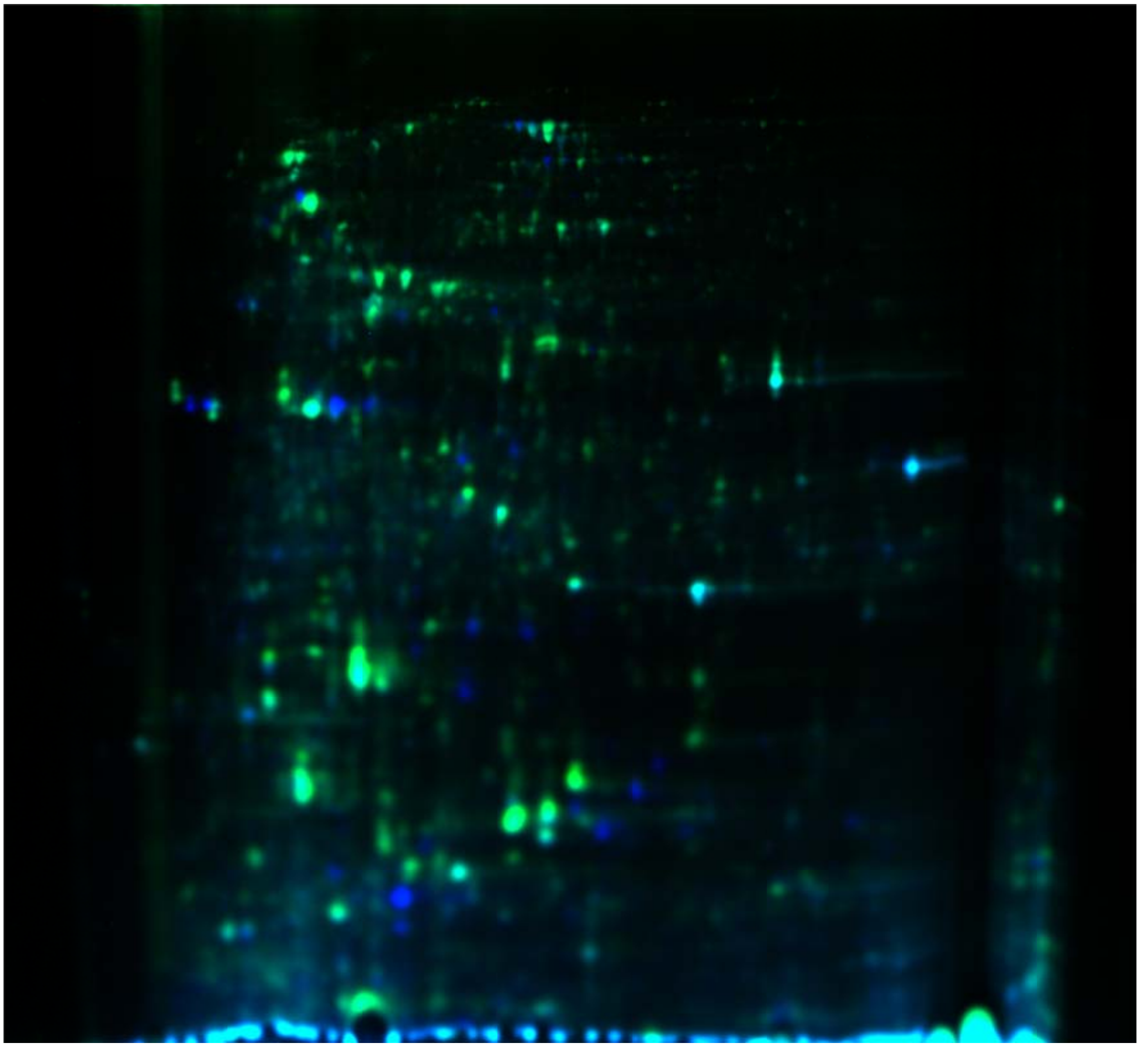

Gel 4 Cy2 vs 07 Cd N2(Cy5)

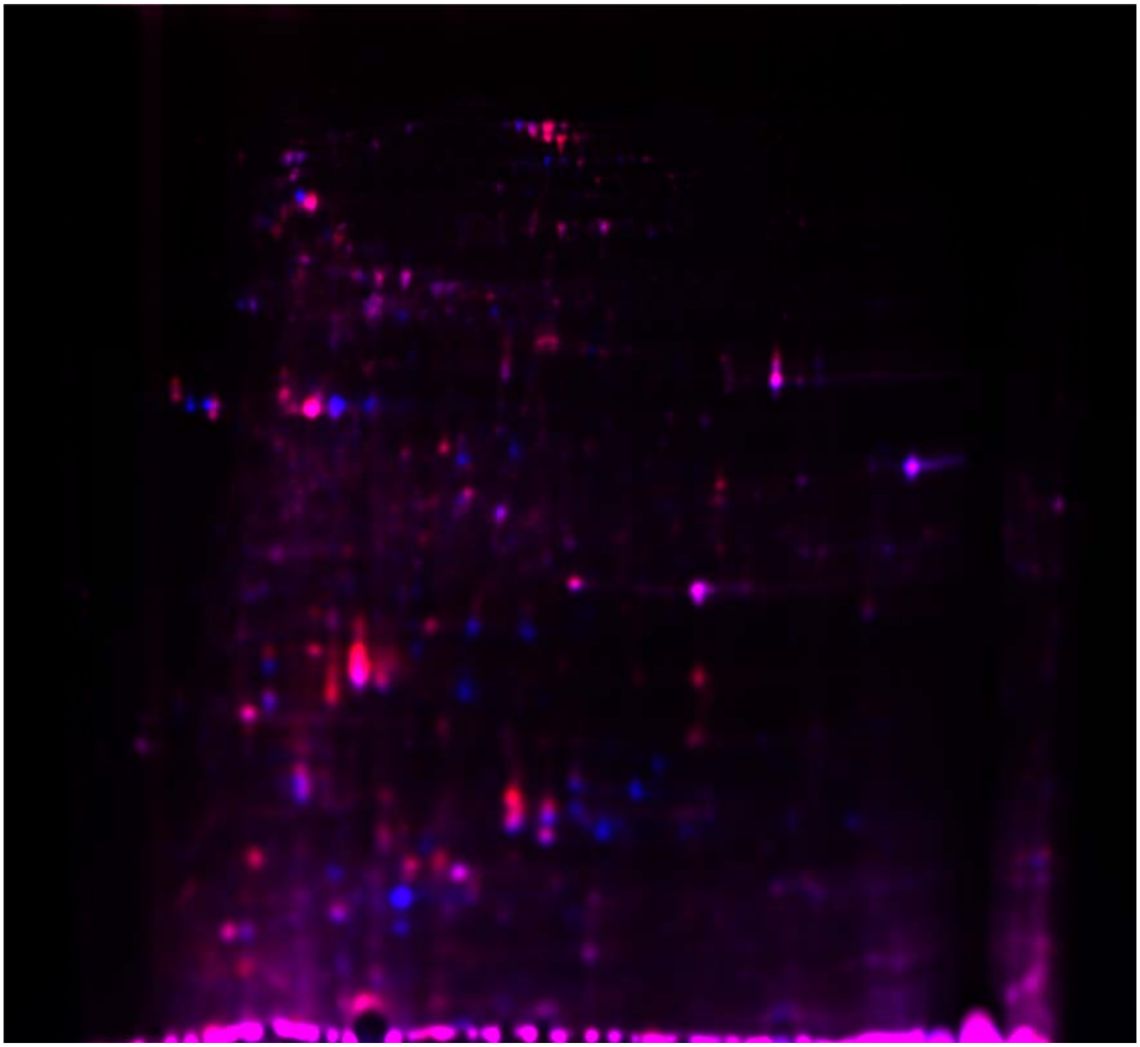

Gel 4 07 Ctrl N2(Cy3) vs 07 Cd N2(Cy5)

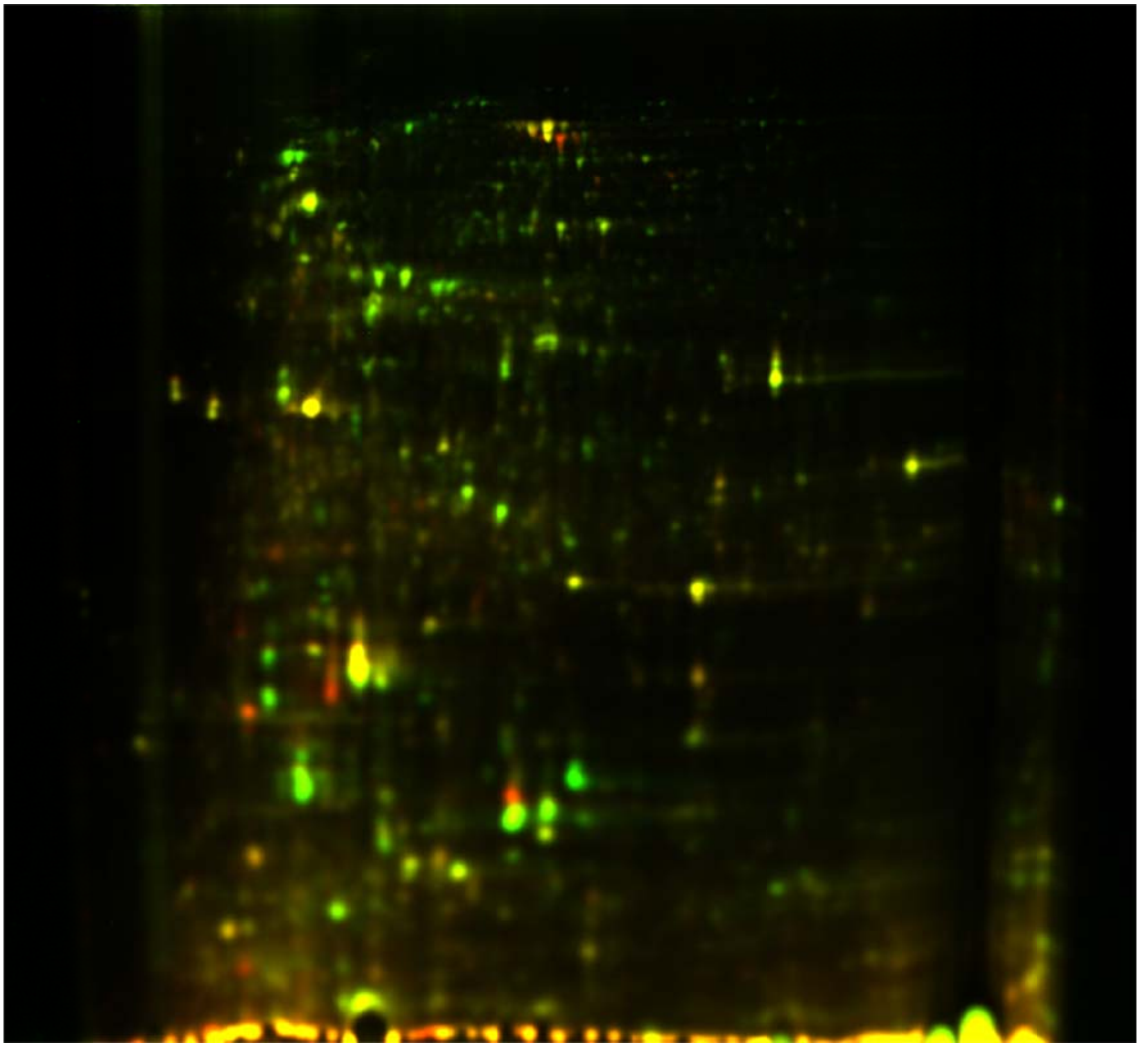

Gel 5 Cy2 vs ATCC Ctrl N3(Cy3) vs 07 Ctrl N1(Cy5)

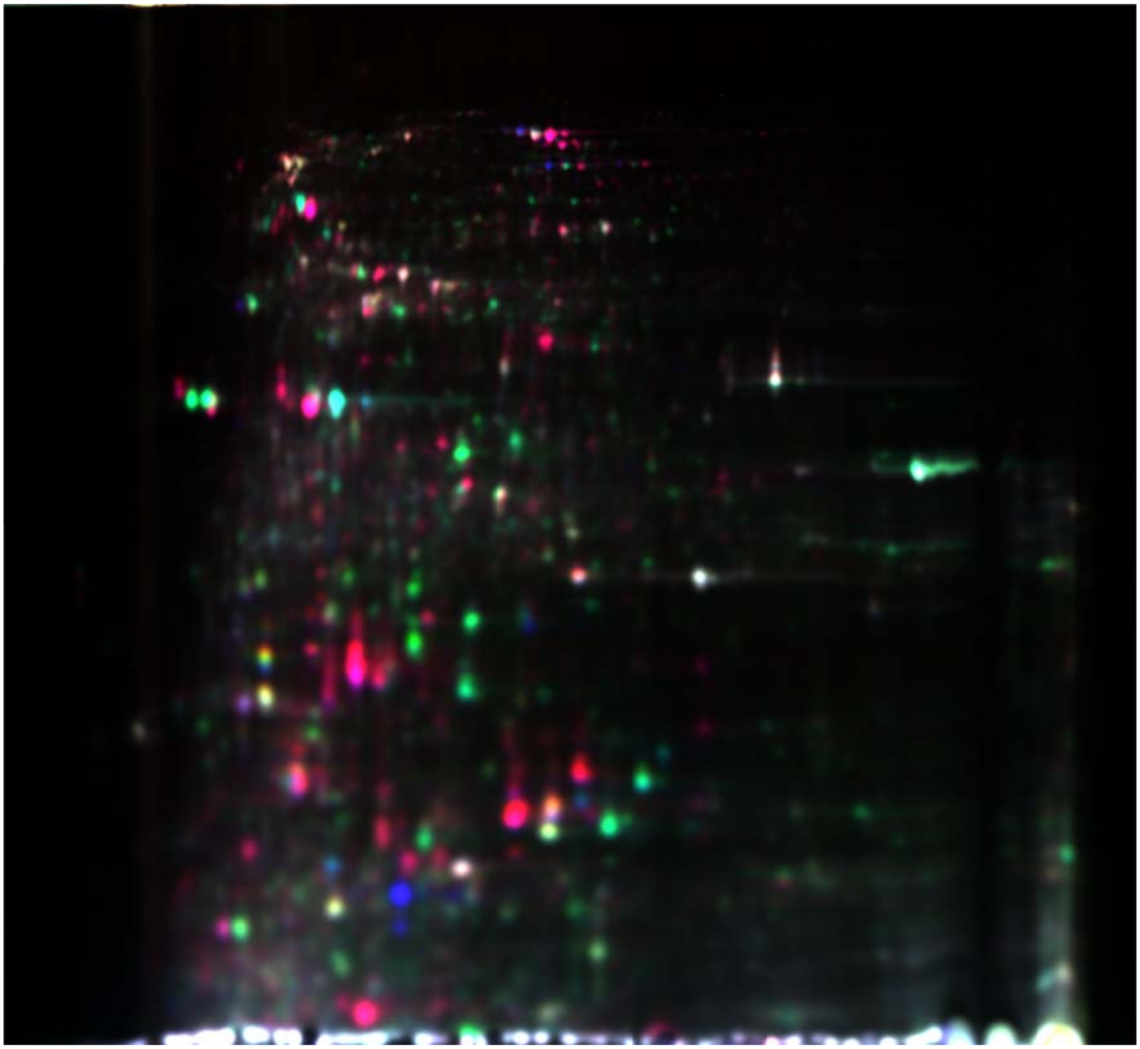

Gel 5 Cy2 vs ATCC Ctrl N3(Cy3)

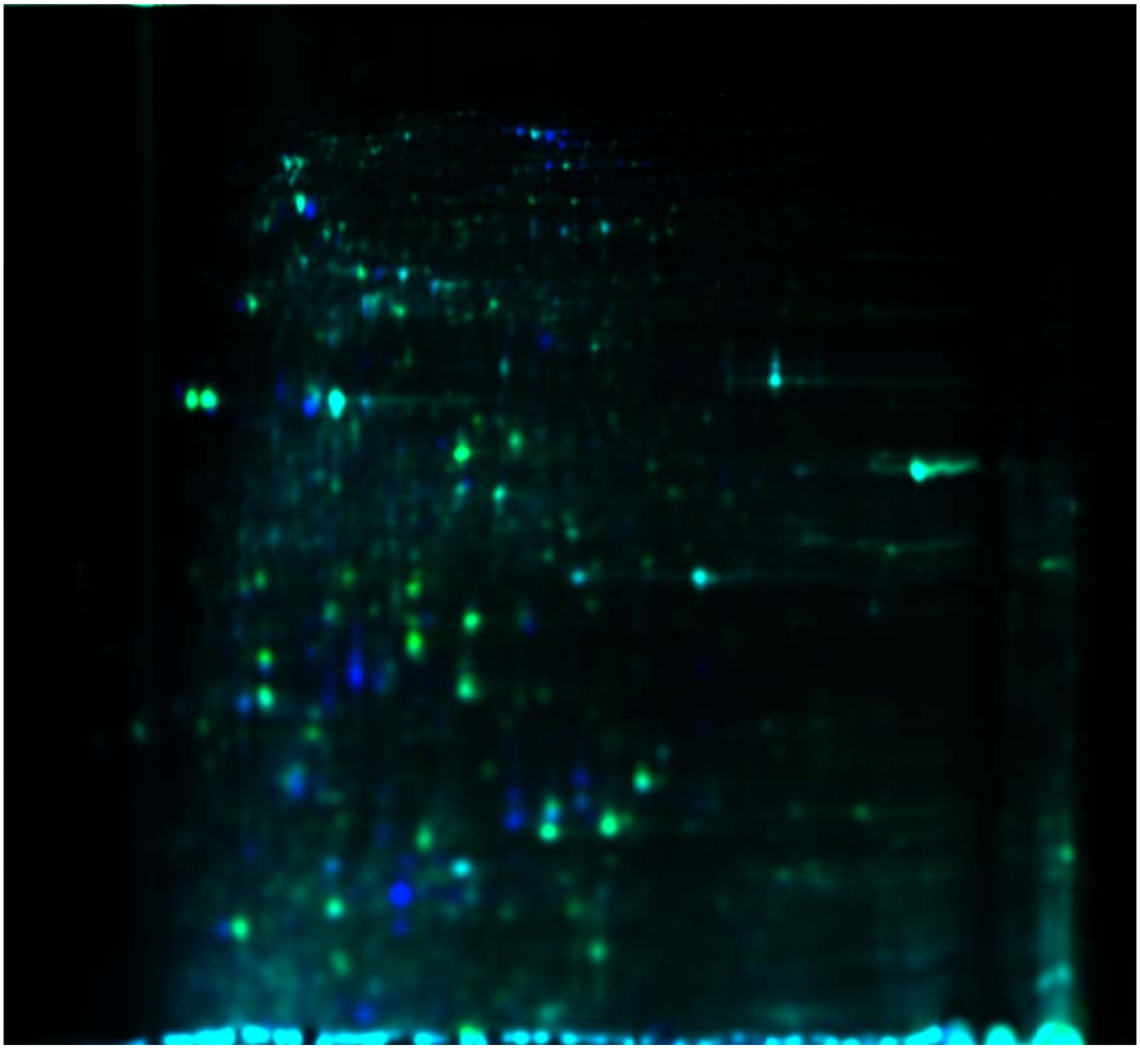

Gel 5 Cy2 vs 07 Ctrl N1(Cy5)

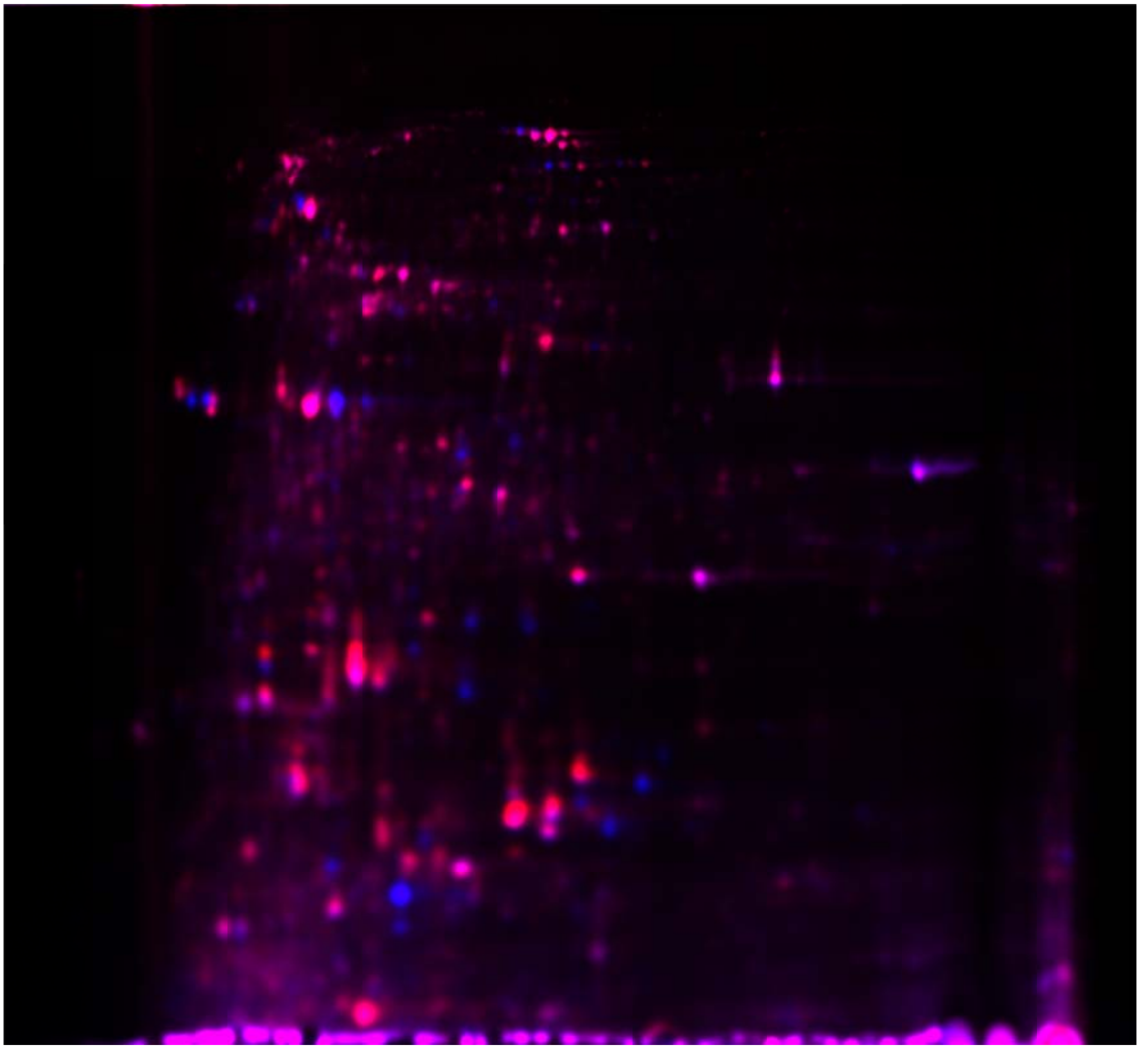

Gel 5 ATCC Ctrl N3(Cy3) vs 07 Ctrl N1(Cy5)

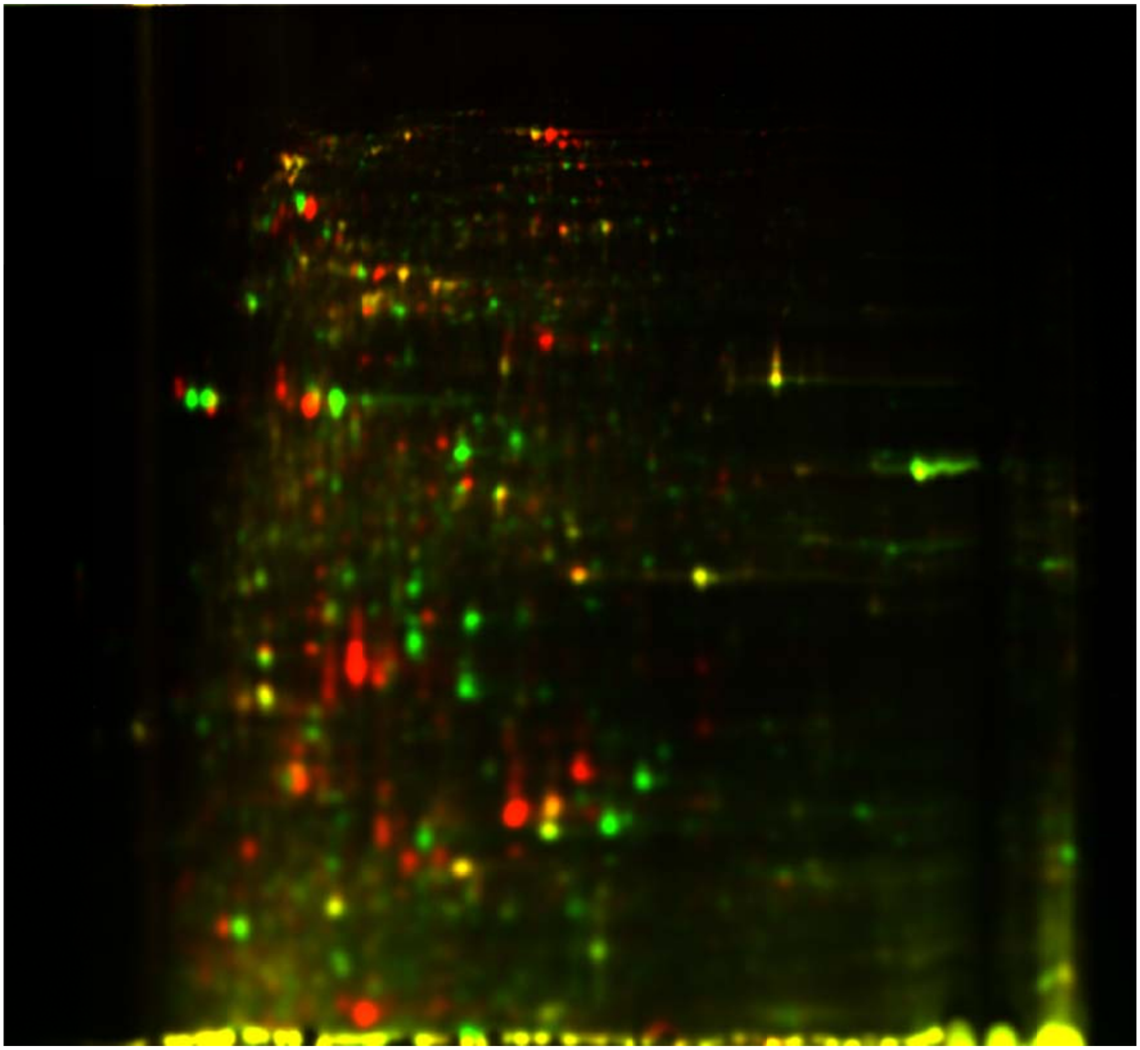

Gel 6 Cy2 vs 07 Cd N3(Cy3) vs 01 Ctrl N3(Cy5)

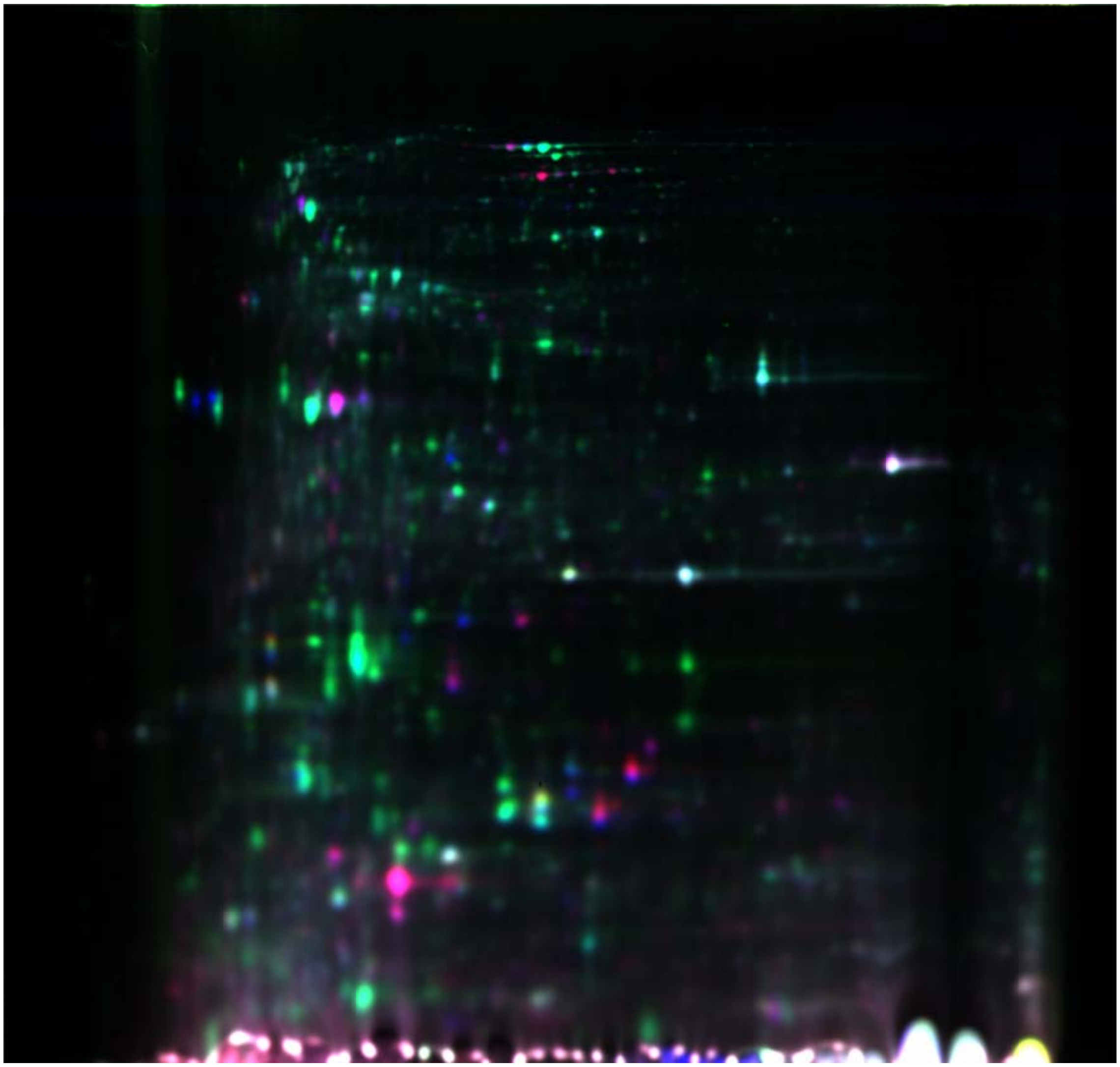

Gel 6 Cy2 vs 07 Cd N3(Cy3)

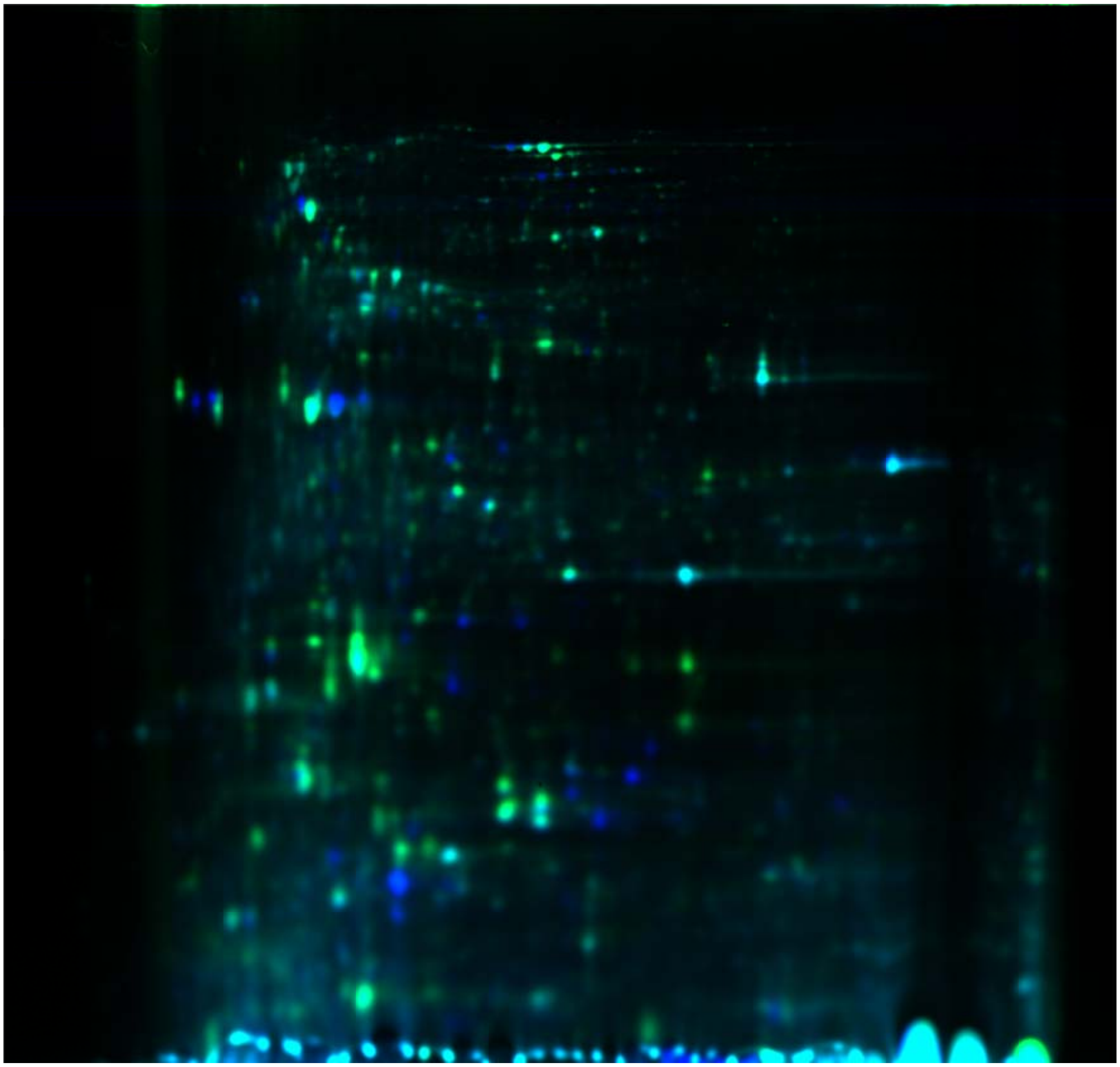

Gel 6 Cy2 vs 01 Ctrl N3(Cy5)

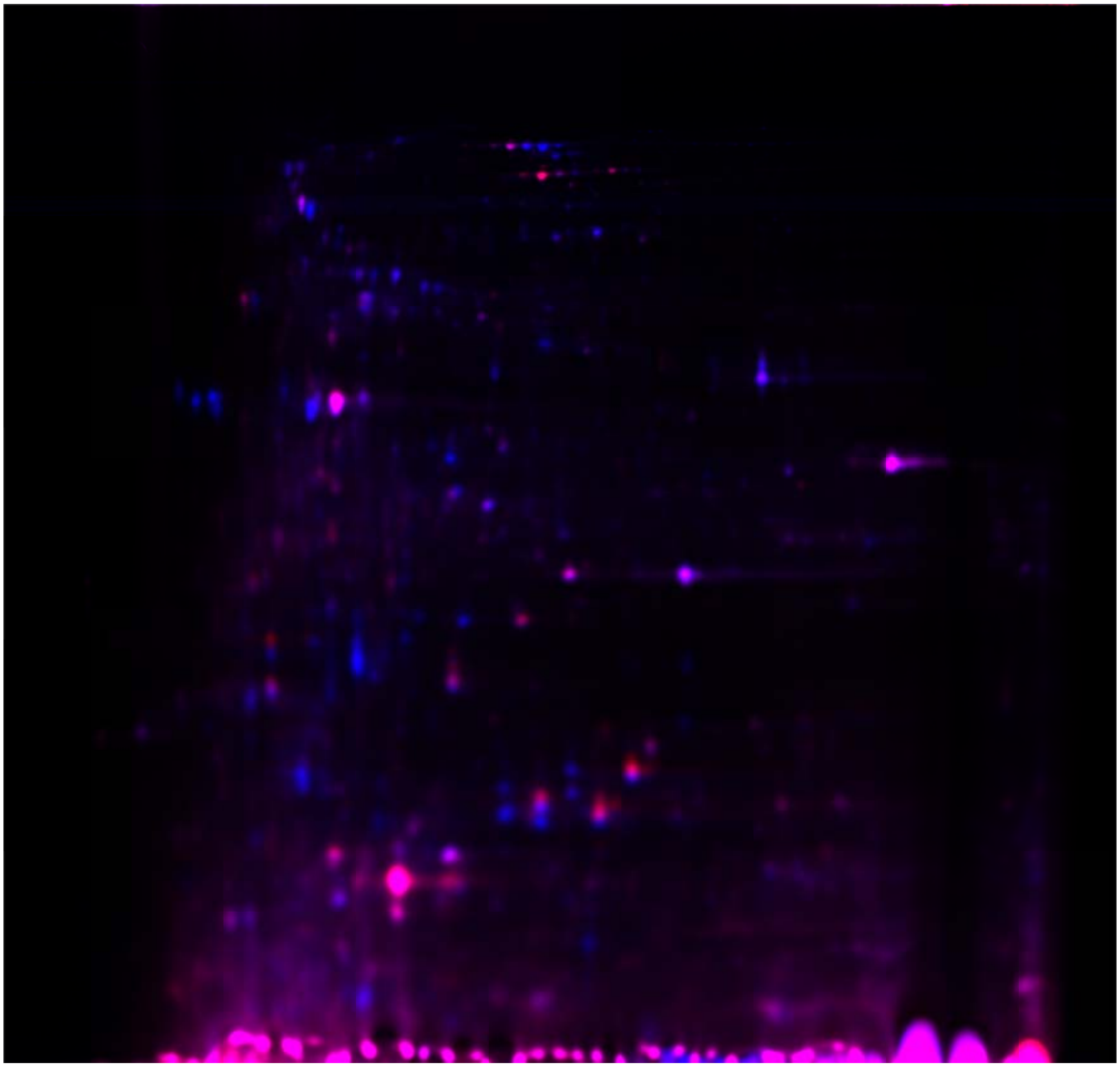

Gel 6 07 Cd N3(Cy3) vs 01 Ctrl N3(Cy5)

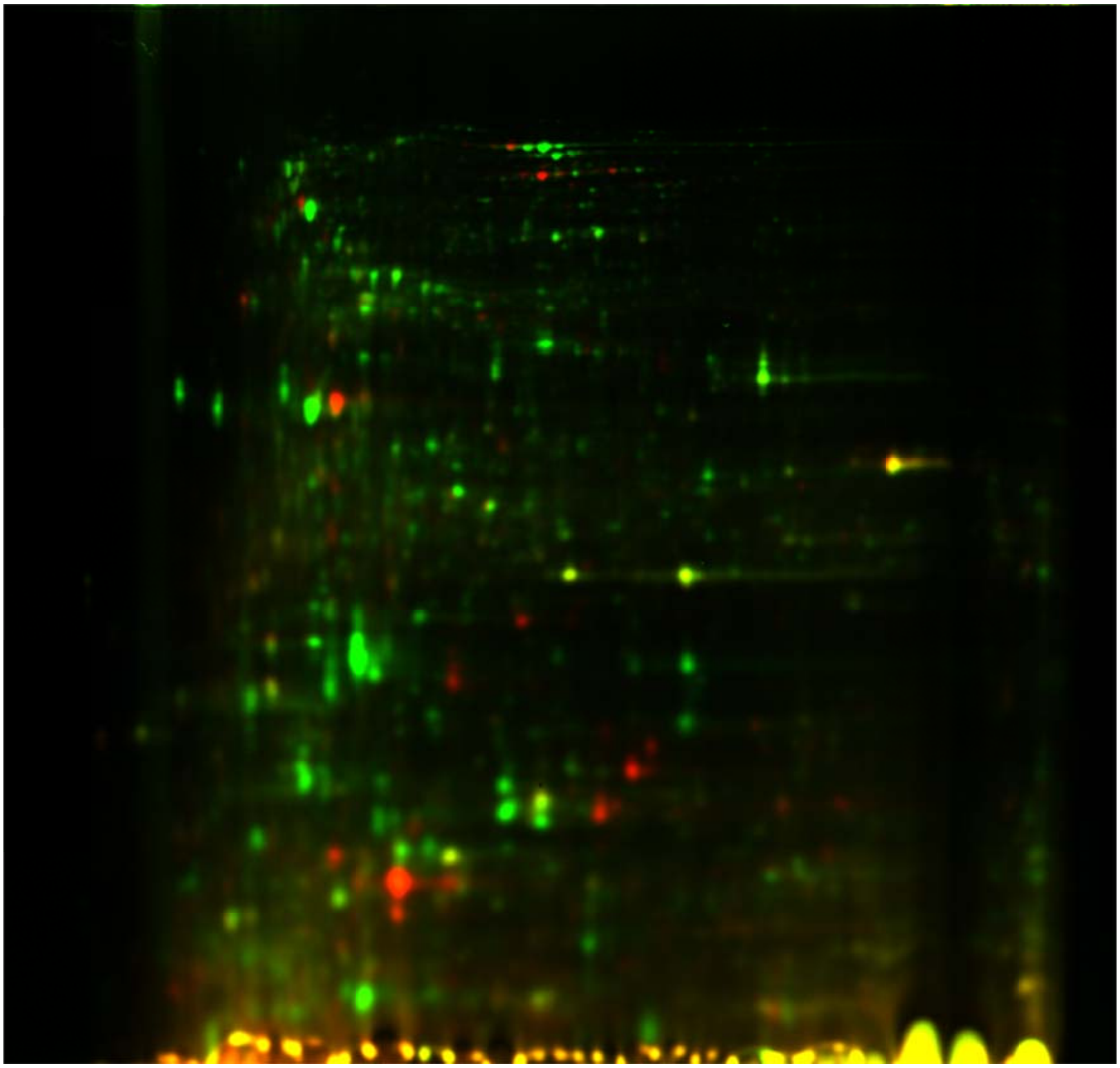

Gel 7 Cy2 vs ATCC Ctrl N2(Cy3) vs ATCC Cd N2(Cy5)

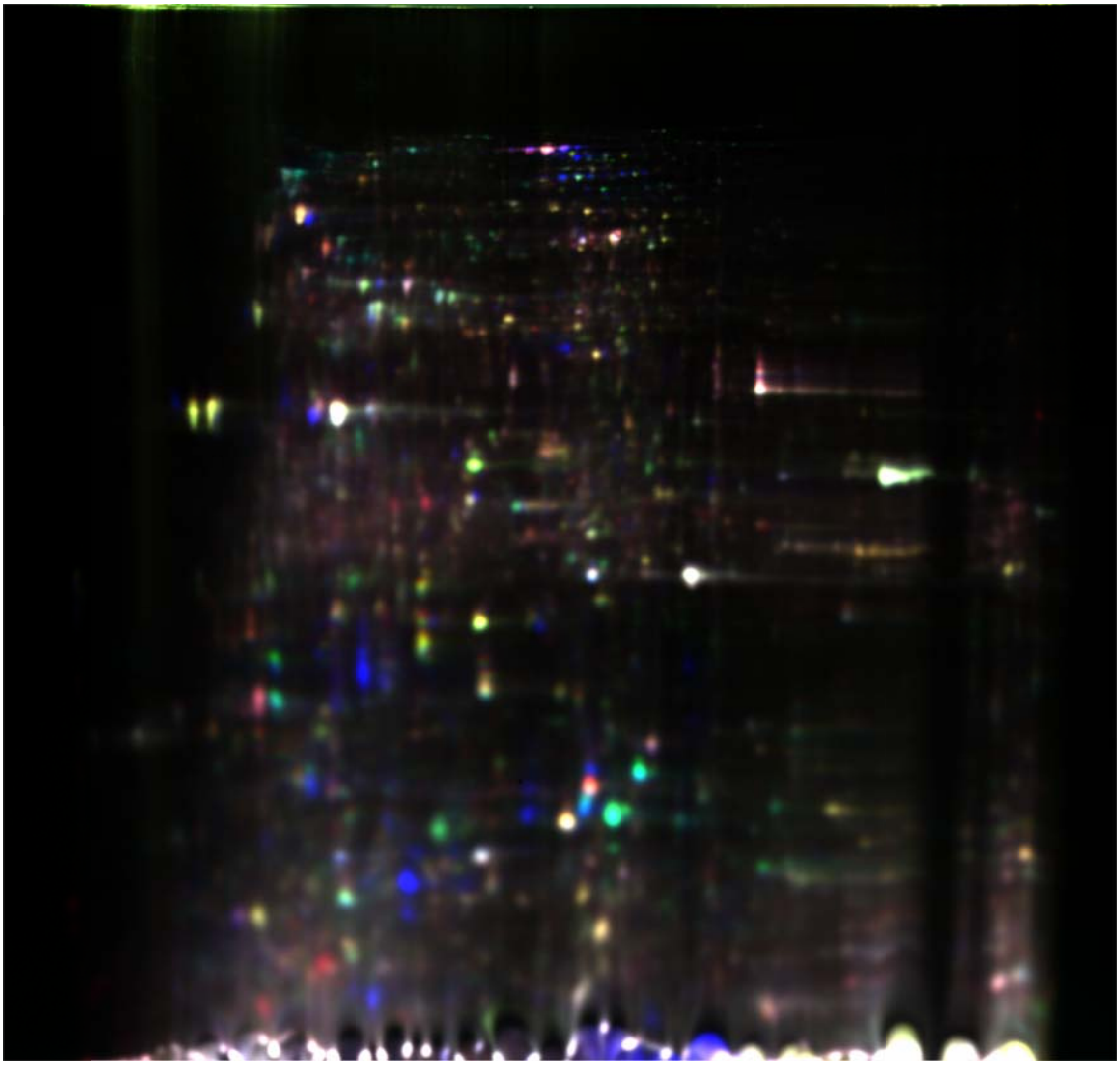

Gel 7 Cy2 vs ATCC Ctrl N2(Cy3)

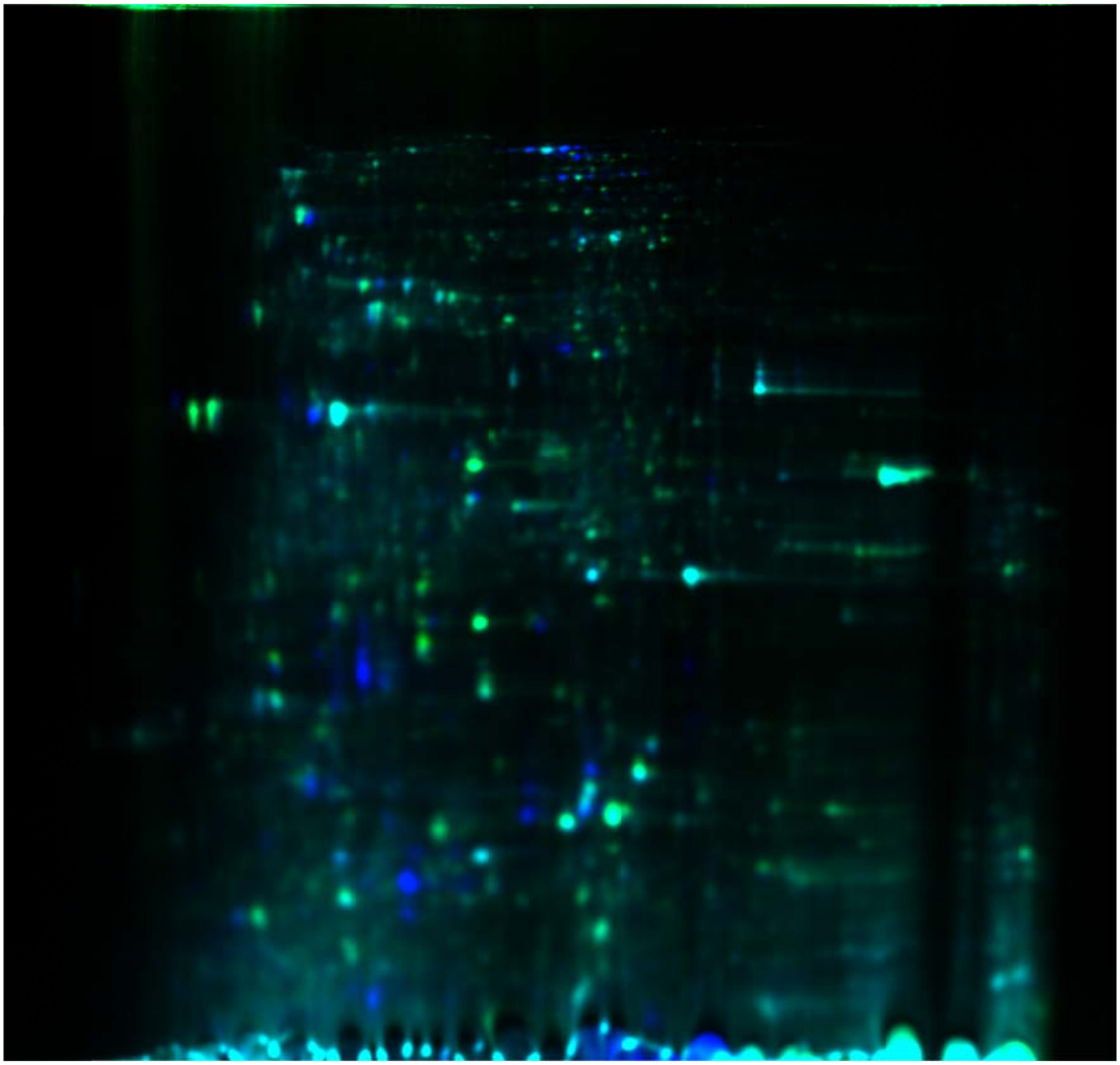

Gel 7 Cy2 vs ATCC Cd N2(Cy5)

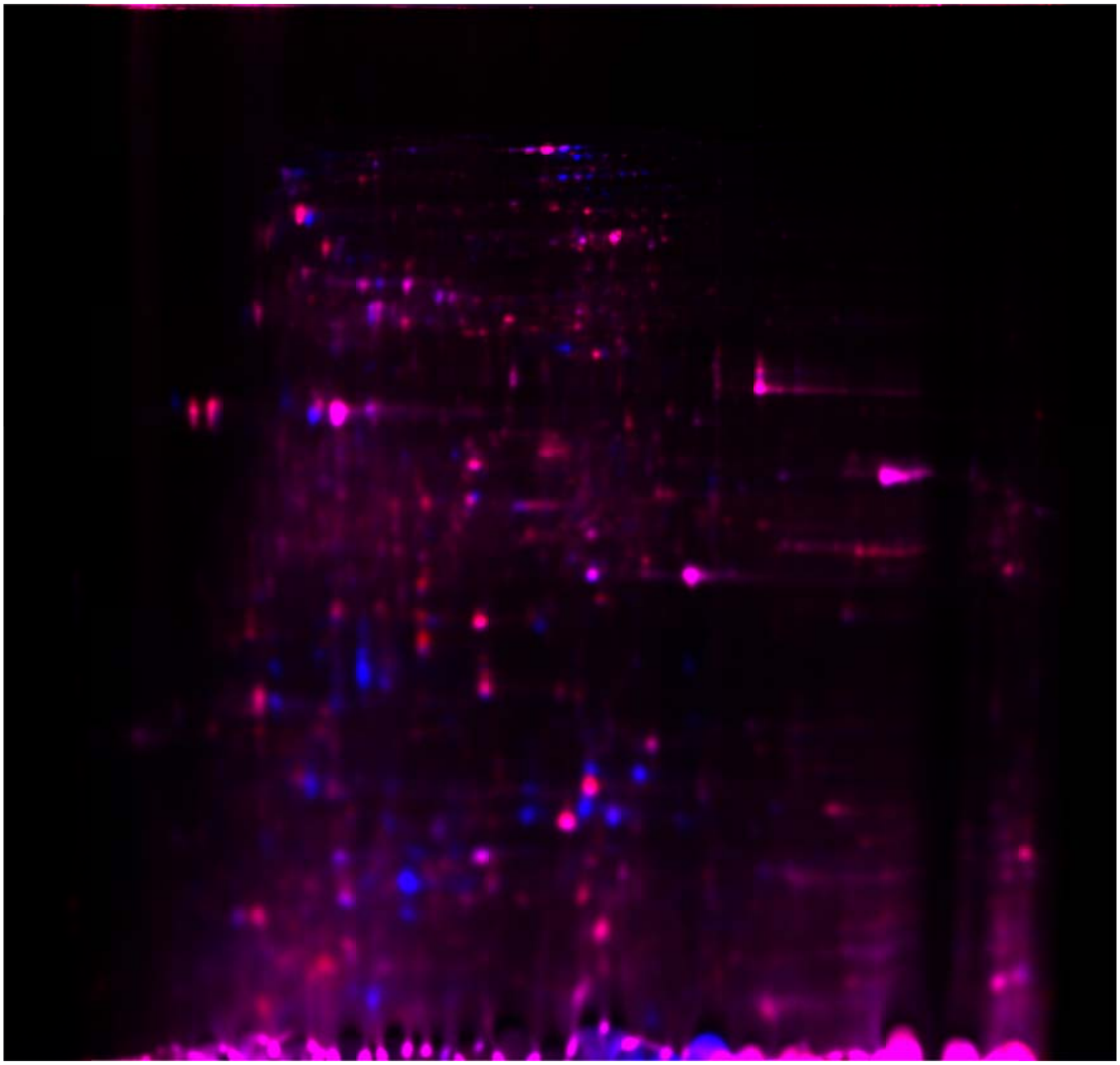

Gel 7 ATCC Ctrl N2(Cy3) vs ATCC Cd N2(Cy5)

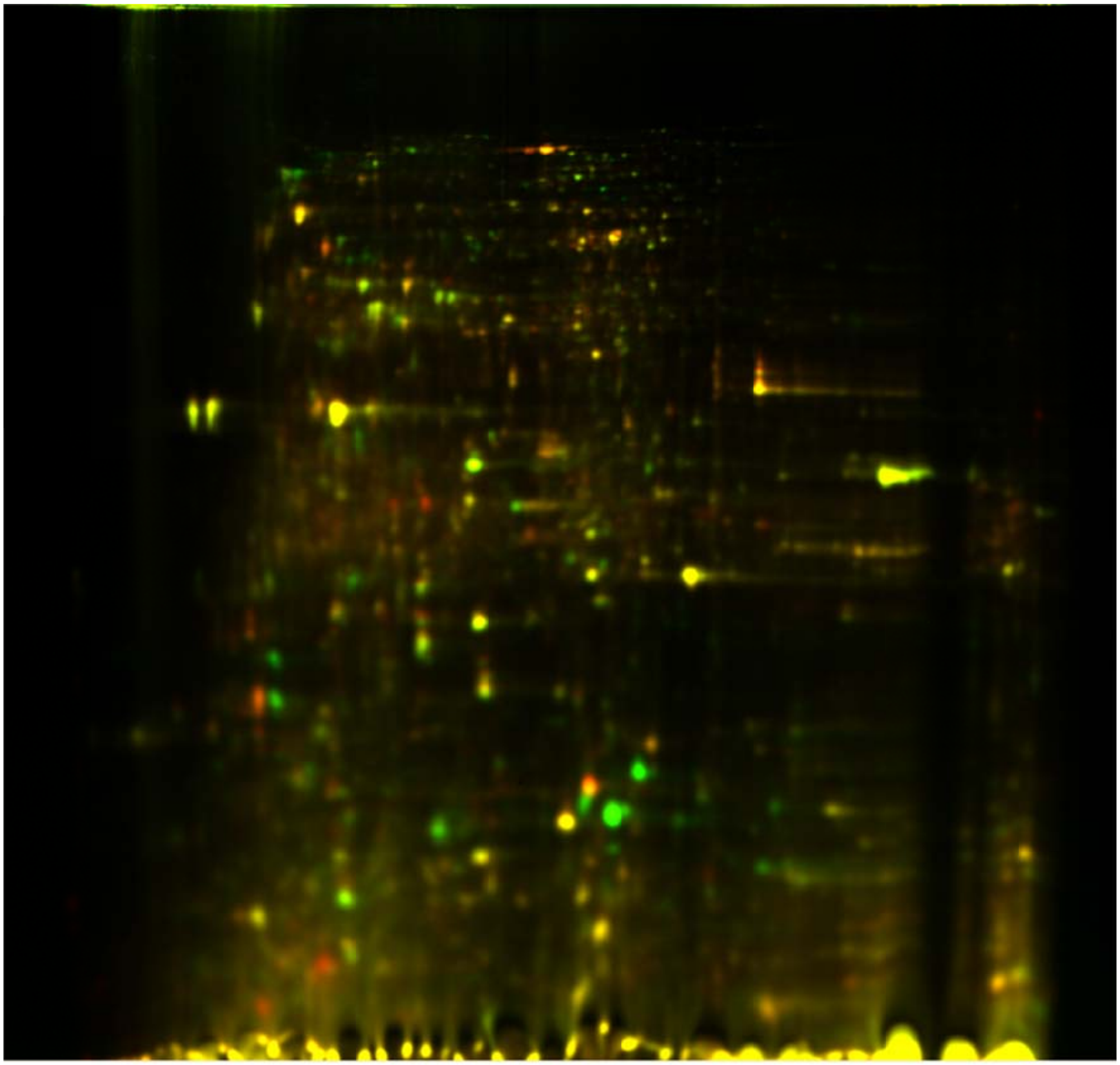

Gel 8 Cy2 vs ATCC Cd N1(Cy3) vs 01 Cd N1(Cy5)

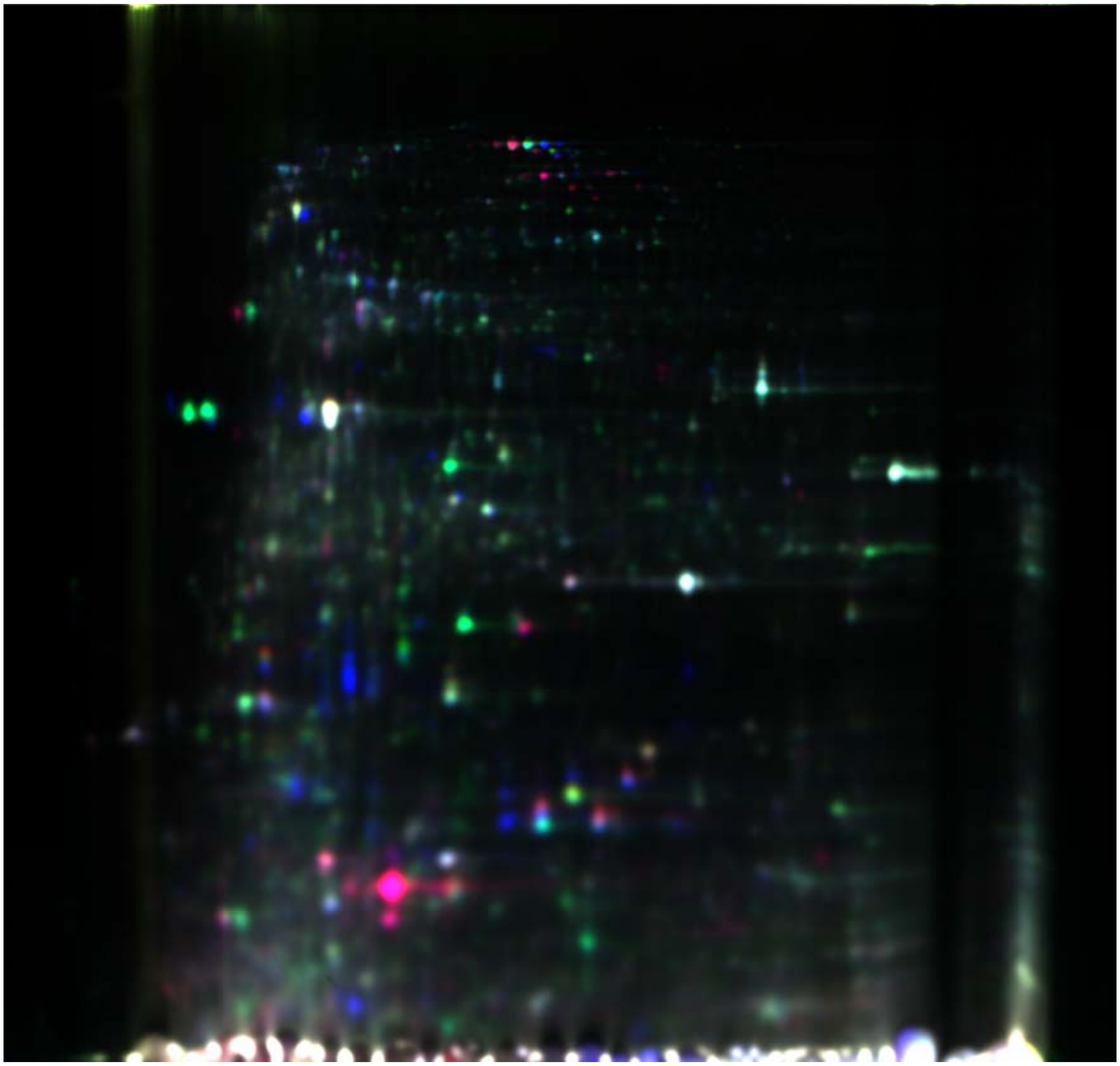

Gel 8 Cy2 vs ATCC Cd N1(Cy3)

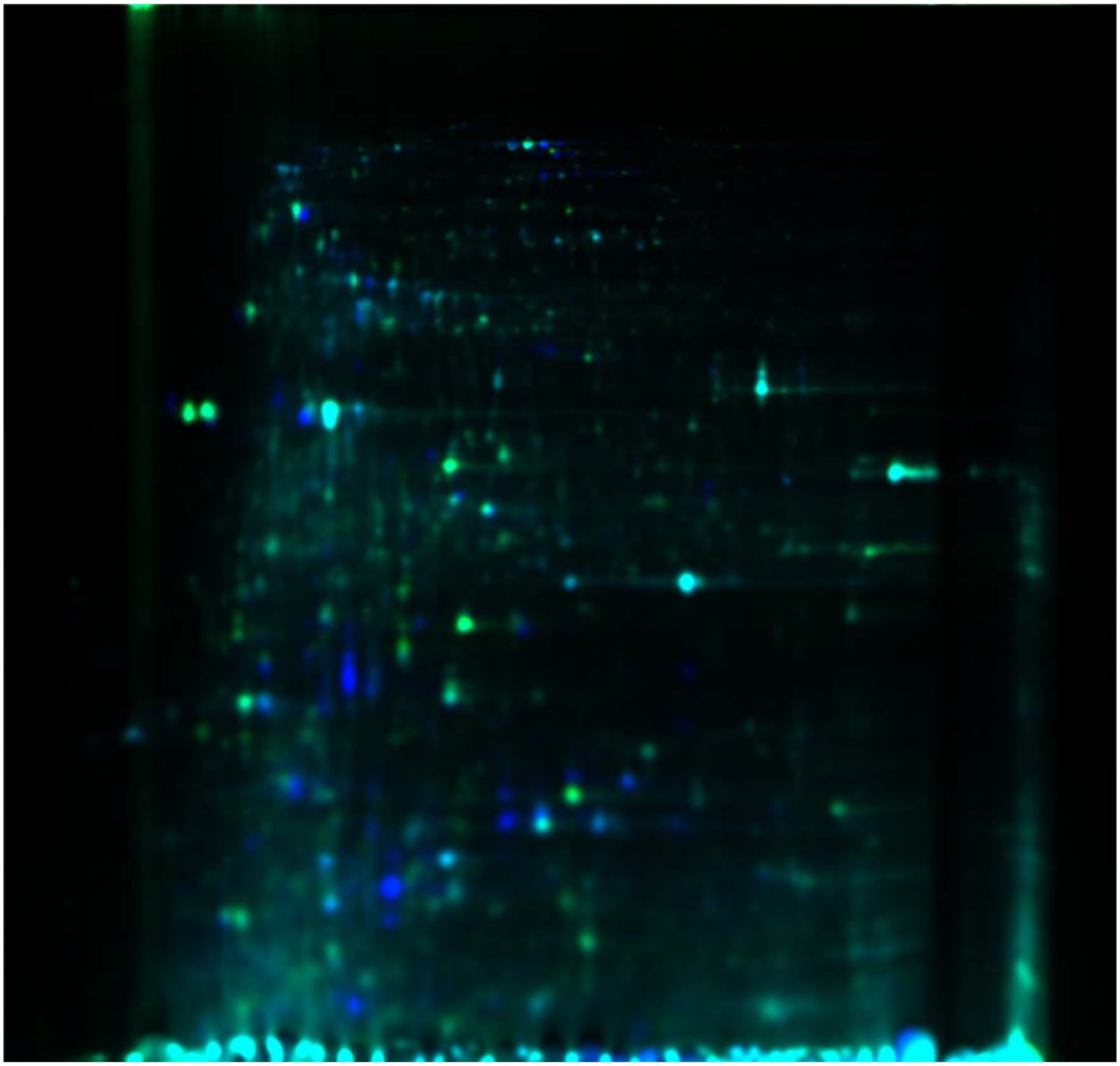

Gel 8 Cy2 vs 01 Cd N1(Cy5)

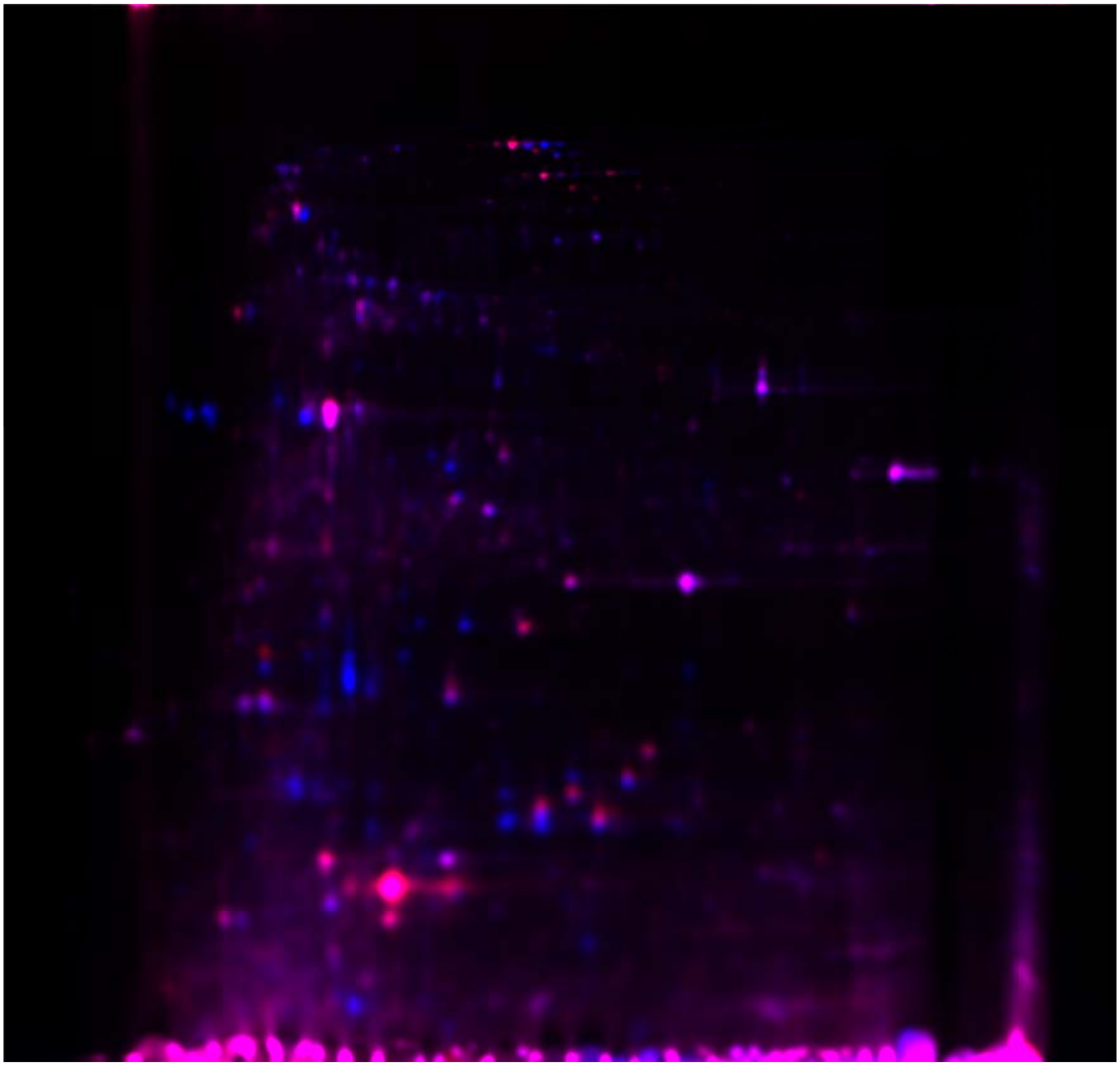

Gel 8 ATCC Cd N1(Cy3) vs 01 Cd N1(Cy5)

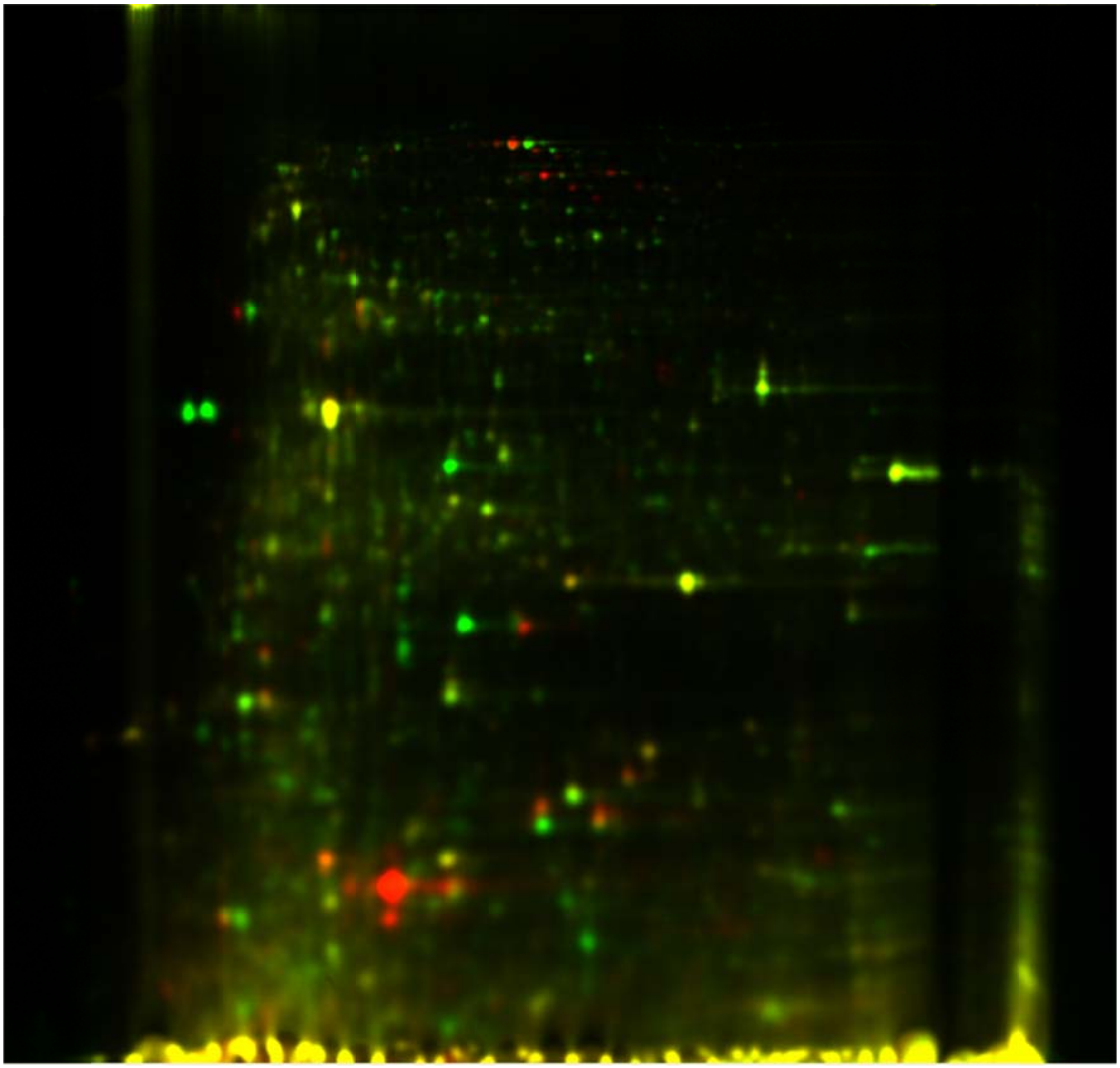

Gel 9 Cy2 vs 01 Cd N3(Cy3) vs ATCC Cd N3(Cy5)

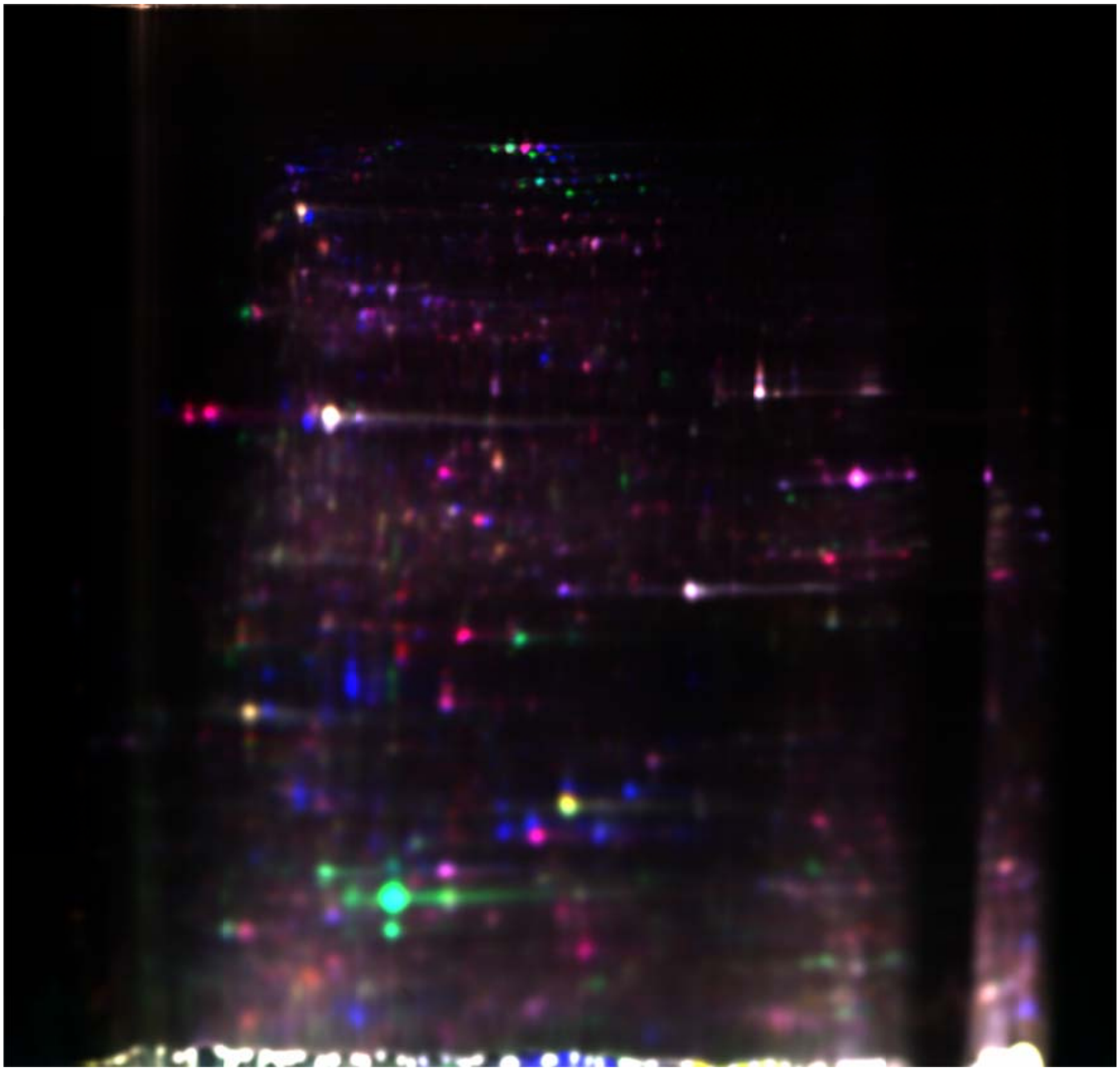

Gel 9 Cy2 vs 01 Cd N3(Cy3)

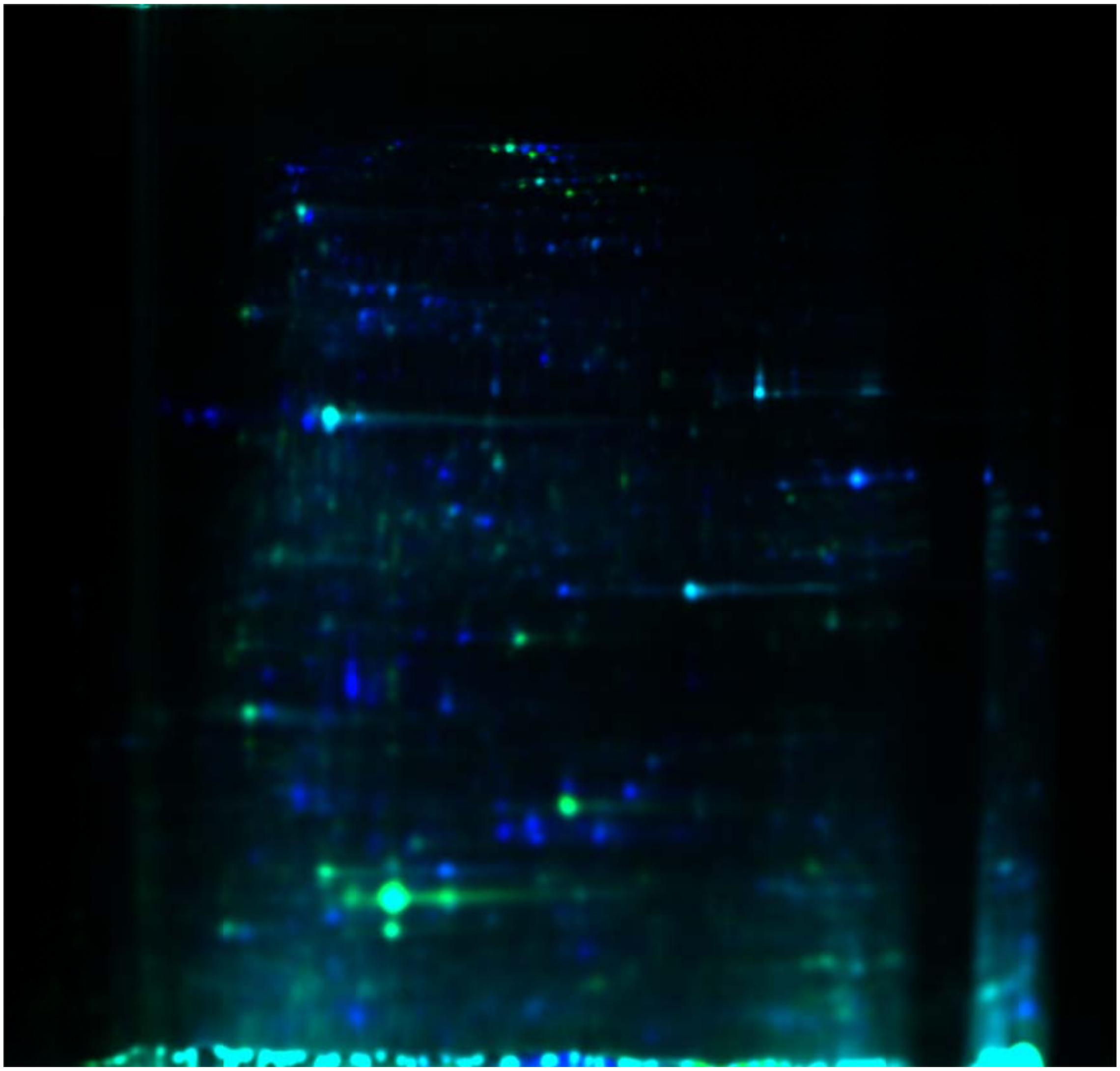

Gel 9 Cy2 vs ATCC Cd N3(Cy5)

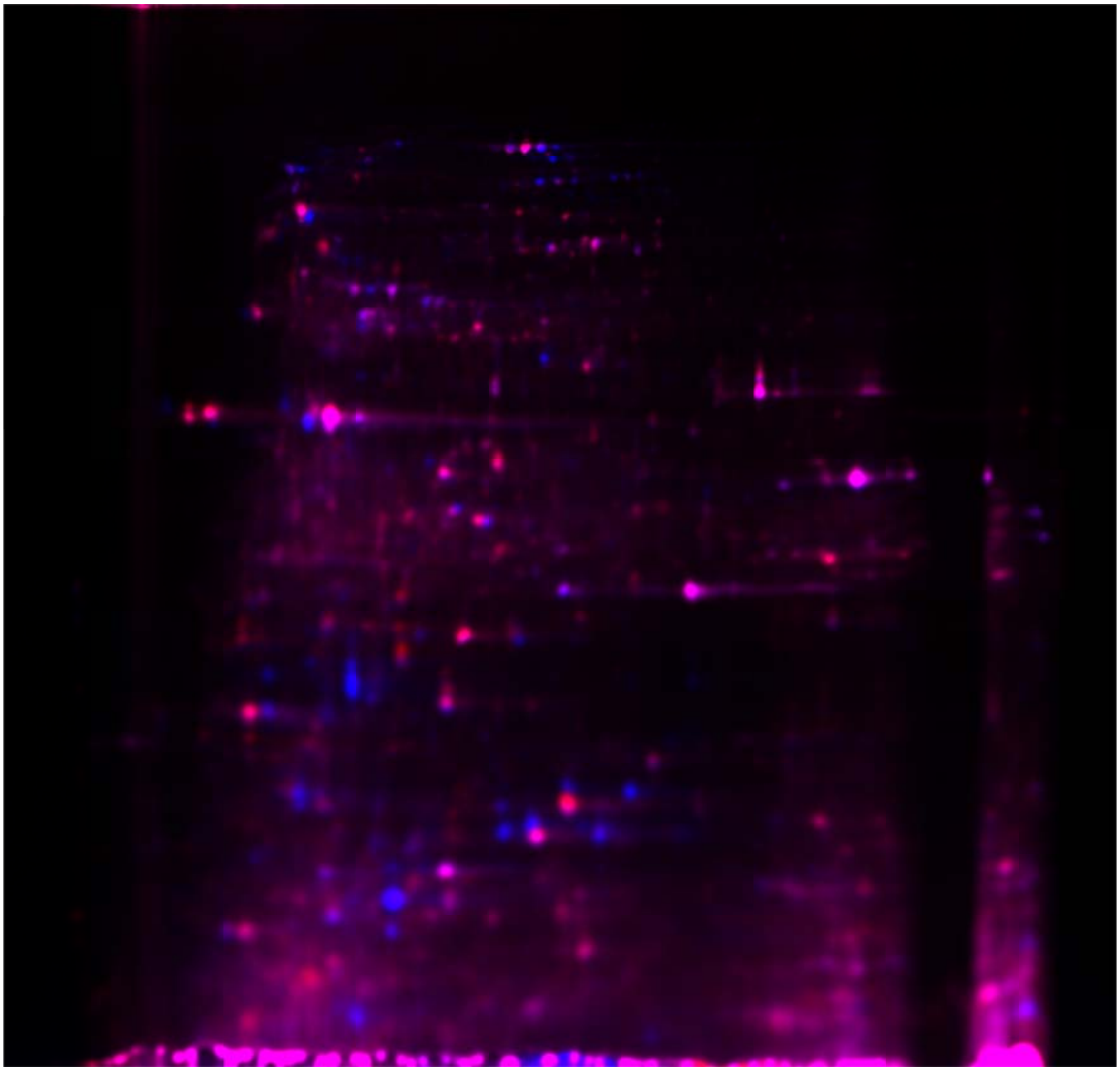

Gel 9 01 Cd N3(Cy3) vs ATCC Cd N3(Cy5)

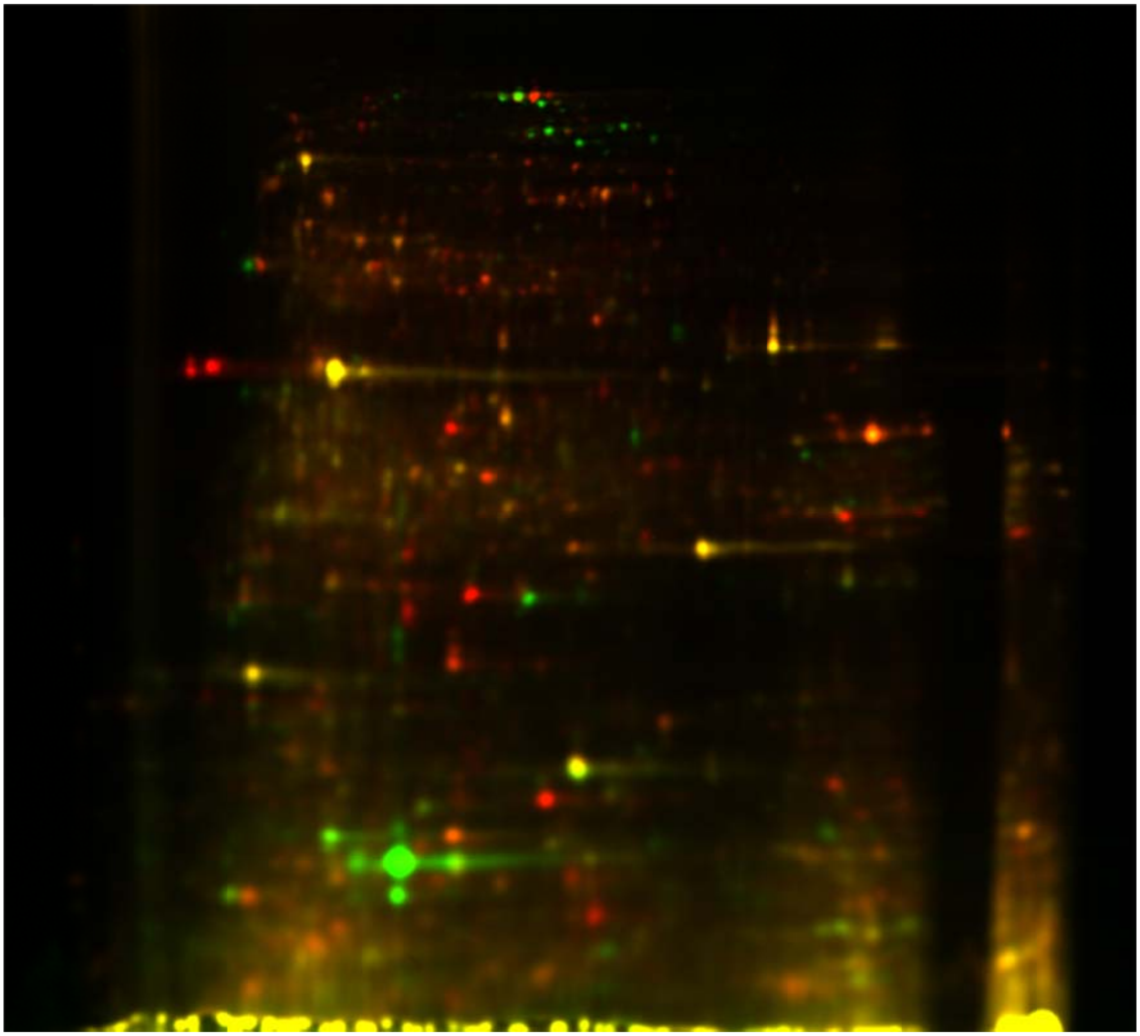

Supplement: Supplemental Information 6 [file peerj-07-6904-s006.pdf]
